# A Pollen‐Enhanced Bionic Mechanoreceptor Induced by Asymmetric Ionic Convection in Hydrogel for Sensory‐Augmented Prostheses

**DOI:** 10.1002/advs.202521235

**Published:** 2026-06-19

**Authors:** Zi Hao Guo, Jingyu Deng, Yanzhang Xu, Chenchen Zhou, Yangshi Shao, Xiong Pu, Munho Kim, Namjoon Cho

**Affiliations:** ^1^ School of Electrical and Electronic Engineering Nanyang Technological University Singapore Singapore; ^2^ School of Materials Science and Engineering Nanyang Technological University Singapore Singapore; ^3^ Centre For Cross Economy Nanyang Technological University Singapore Singapore; ^4^ Beijing Institute of Nanoenergy and Nanosystems Chinese Academy of Sciences Beijing P. R. China

**Keywords:** bioinspired, bionic, ionic devices, mechanoreceptors, plant‐based, pollen, prosthesis

## Abstract

The growing prevalence of age‐related limb loss underscores the need for prosthetic technologies that restore not only motor function but also authentic sensory feedback. Current prosthetic systems largely depend on sensory substitution or signal remapping, which fall short of replicating natural somatosensory signals. In this work, we develop a plant‐enhanced bionic mechanoreceptor that mimics biological touch by converting mechanical stimuli into ionic signals. Incorporating bio‐derived pollen microgels into the hydrogel matrix introduces interfacial ion‐anchoring sites that strengthen cation–matrix interactions, enhance ionic polarization, and significantly amplify the piezoionic output. This enhancement arises from pressure‐driven asymmetric ion transport within the ionically conductive hydrogel. As a result, the output signal increases by up to 12‐fold, providing a simple and accessible strategy to improve the sensitivity of piezoionic mechanoreceptors. Then, we demonstrate the integration of ten such mechanoreceptors into a robotic prosthetic arm and utilize a deep learning algorithm to interpret the complex signal patterns. The system achieves accurate recognition of object interaction, validating the potential for naturalistic tactile feedback. This platform offers a scalable, biomimetic solution for developing next‐generation sensory‐augmented prostheses and may inform future designs in neuroprosthetics and human–machine interfaces.

## Graphical abstract

This work proposed a pollen‐enhanced bionic mechanoreceptor based on ionic convection. Leveraging the ion anchoring effect of the pollen particle, the output performance could be ∼12 times higher than the original state. By employing deep learning models as AI brains, the feasibility of a sensory‐augmented prosthesis consisting of a pollen‐enhanced bionic mechanoreceptor was also verified.

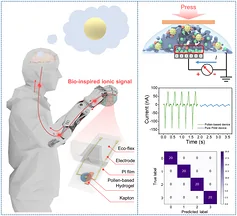

## 1 Introduction

**FIGURE 1:**
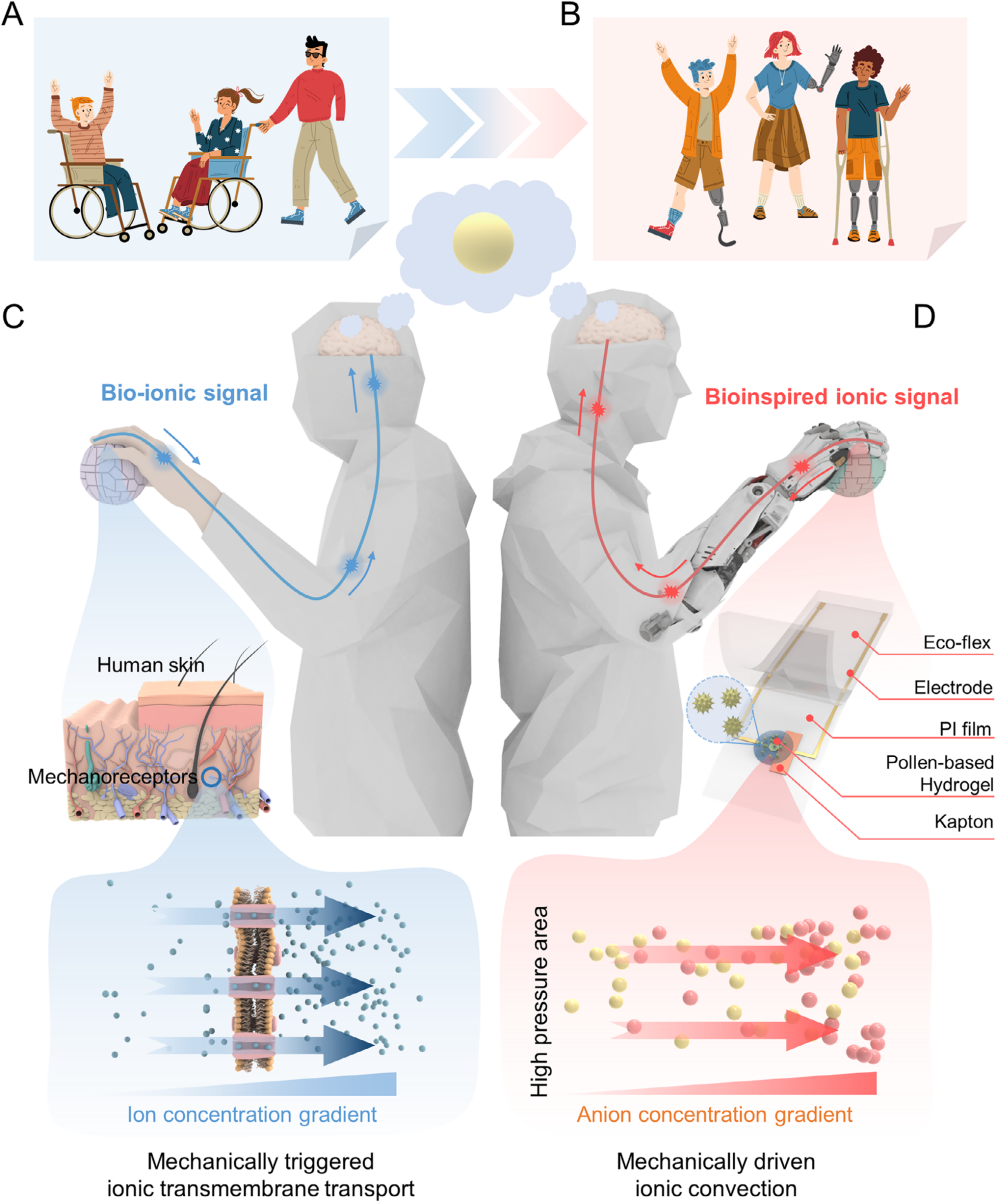
Schematic illustration of the bioinspired strategy to develop the sensory‐augmented prostheses. (A) Disables with the loss of limbs. (B) Wearing prosthetic devices is the most efficient way for people with limb deficiencies to bring them back to normal life. To provide real feedback signals to the user, a plant‐enhanced piezoionic mechanoreceptor was developed by mimicking the mechanoreceptor in human skin. (C) The mechanism of bio‐ionic signal generation from skin mechanoreceptors which is mainly based on mechanically triggered ionic transmembrane transport. (D) The structure of plant‐enhanced bionic mechanoreceptor and the bioinspired ionic signal generation process that based on mechanically driven ionic convection.

The ability to perceive and respond to mechanical stimuli is a fundamental aspect of human interaction with the physical environment [1, 2, 3]. Tactile sensation, in particular, plays a central role in spatial perception, motor coordination, and daily functionality, enabling precise manipulation and engagement with our surroundings [4, 5]. Yet, as the aging population has increased, the burden of sensory and limb impairments is escalating at an alarming pace [6, 7, 8]. Age‐related neurodegenerative diseases, traumatic injuries, and chronic conditions are contributing to a surge in individuals experiencing diminished or lost sensory function [9, 10, 11, 12]. The World Health Organization (WHO) estimates that over 1.3 billion people—representing 16% of the global population—live with significant disabilities, underscoring a profound and growing challenge for public health and technological innovation alike [13]. Addressing this issue calls for a reimagining of human–machine interfaces that can restore, augment, or even transcend the limitations of the human sensory system [1, 14]. Individuals with physical disabilities, particularly those with limb deficiencies, experience a substantial reduction in their quality of life due to the loss of natural sensory and motor functions. The use of prosthetic devices represents one of the most effective strategies for restoring functional independence and enhancing daily well‐being (Figure 1A,B) [15, 16, 17].

Modern prosthetic devices for amputees are primarily engineered to establish reliable bidirectional human–machine interfaces that can restore sensorimotor control [18, 19, 20]. In particular, for the sensory afferent pathway, it is critical to transmit peripheral sensory information to the central nervous system (CNS) [21, 22]. Although the native mechanoreceptors are lost along with the amputated limb, residual peripheral nerves often remain intact and can still relay sensory signals to the CNS [19].

To exploit these pathways, noninvasive feedback techniques such as sensory substitution or remapping—employing modalities like vibration, pressure, or low‐level electrical stimulation—are commonly utilized to improve object manipulation and enhance user experience during prosthesis operation [23, 24, 25, 26]. However, these approaches fail to replicate the native mechanisms of afferent sensation, providing surrogate rather than naturalistic feedback.

Biological systems, however, offer ideal templates to address these challenges. Mechanoreceptors embedded within the skin naturally transduce mechanical stimuli into intracellular bio‐ionic signals via ionic transmembrane transport (Figure 1C), forming the fundamental mechanism of tactile perception [27]. When pressure is applied to the skin, sodium ions influx into the cell membrane, increasing the membrane potential. Subsequently, voltage‐gated ion channels open, permitting reverse ion transport and generating alternating bio‐ionic signals that convey tactile information to the nervous system.

In this work, inspired by the mechanoreceptors in human skin, we developed a planar‐shaped bionic mechanoreceptor to generate a bioinspired ionic signal through asymmetric mechanically driven ionic convection (Figure 1D). The device generates an output of ∼10 mV and ∼12 nA, indicating its potential suitability for neuro‐prosthetic signal interfacing. For the sensory substitution strategy, the applied electrical stimuli should be several microamperes [28]. In contrast, if electrical stimuli are applied directly to peripheral nerves, even nanoampere‐level currents are sufficient to elicit action potentials [29]. Furthermore, clinical evidence suggests that the perceived intensity of stimulation is generally positively correlated with the output current of external rehabilitation devices [28]. To enhance the output of devices that are based on ionic‐induced polarization, e.g., piezoionic devices, iontronic flexoelectric devices, mechanoionic asymmetry hydrogel generators, etc., surface modification or hydrogel matrix molecular chain modification methods are reported [29, 30, 31]. However, existing strategies to enhance ionic polarization‐based devices often require complex chemical fabrication processes, limiting their scalability and practical deployment. To addres this challenges, sunflower pollen particles were incorporated into the hydrogel matrix, creating a heterogeneous ionic environment. The presence of these plant‐derived particles imposes spatial confinement and induces electrostatic interactions that selectively impede cation mobility relative to anions. This ion transport asymmetry significantly amplifies ionic polarization during mechanical deformation along the compression axis, resulting in an approximately 12‐fold enhancement in output current, reaching up to 143.4 nA. Moreover, the ionic current generated by the plant‐enhanced bionic mechanoreceptor can be readily modulated across a wide range, offering tunability to accommodate diverse application scenarios in future sensory‐augmented systems. In contrast to hydrogen‐bond‐triggered ion pumping or trap‐release mechanisms commonly employed in iontronic mechanoreceptors [32, 33, 34], our approach enhances bulk piezoionic transport asymmetry within an aqueous hydrogel through interfacial cation anchoring. This approach eliminates the need for ionic liquids, microengineered pumping units, or complex nanomaterial synthesis.

We hypothesize that our plant particle‐enhanced bionic mechanoreceptor can serve as an external sensory interface on a prosthesis, amplifying ionic signals through enhanced ionic polarization and directly transmitting these signals to the residual peripheral nerves to restore afferent sensory feedback. To validate the concept of a sensory‐augmented prosthesis, we employed a robotic arm as a prototype and integrated ten plant‐enhanced bionic mechanoreceptors onto its surface. These devices were used to collect bioinspired ionic signals generated during the grasping of various objects. To simulate the sensory recognition process, we implemented a deep learning framework as the AI brain. Initially, we utilized a conventional Convolutional Neural Network (CNN), achieving 100% recognition accuracy after 36 training epochs. To further evaluate the system's bioinspired compatibility, we employed a Spiking Neural Network (SNN)—a class of neural networks that more closely mimics the event‐driven processing of biological brains and has been increasingly applied in neuromorphic computing, brain–machine interfaces, and sensory processing. Remarkably, the SNN also achieved 100% recognition accuracy after only 10 training epochs, demonstrating both the robustness and the biological relevance of our sensory interface system.

The high recogenation accuracy indicate that the AI brain recognized different objects based on the bioinspired ionic signals generated by our bionic mechanoreceptors, demonstrating the feasibility of using sensory‐augmented prostheses to deliver physiologically relevant feedback to the nervous system in future applications. Taken together, sensory‐augmented prostheses have the potential not only to enable amputees to perform routine daily tasks, but also to restore precise tactile sensations that closely resemble those of their original limbs.

## 2 Results and Discussion

### 2.1 Structural and Functional Design of the Bionic Mechanoreceptor

The designed structure of the plant‐enhanced bionic mechanoreceptor is schematically shown in Figure 1D. Planar silver electrodes are printed onto a polyimide (PI) film using a Direct Ink Writing (DIW) technique, which offers excellent consistency and holds significant promise for the large‐scale production of these devices (Figure S1a). A cone‐shaped pollen hydrogel composed of sunflower pollen particles embedded within a polyacrylamide (PAM) matrix is positioned between the two electrodes. To ensure that only the central and peripheral regions at the hydrogel's base are electrically connected, a piece of Kapton tape is applied to partially cover the electrode beneath the hydrogel. A thin layer of Ecoflex silicone is used to encapsulate the entire device, providing both protection and sealing to prevent leakage or evaporation of the hydrogel's electrolyte. The detailed fabrication process, along with the device photograph, is shown in Figure S1 and Methods. Notably, excellent flexibility could be observed from the photograph of the as‐fabricated plant‐enhanced bionic mechanoreceptor (Figure S1d).

**FIGURE 2:**
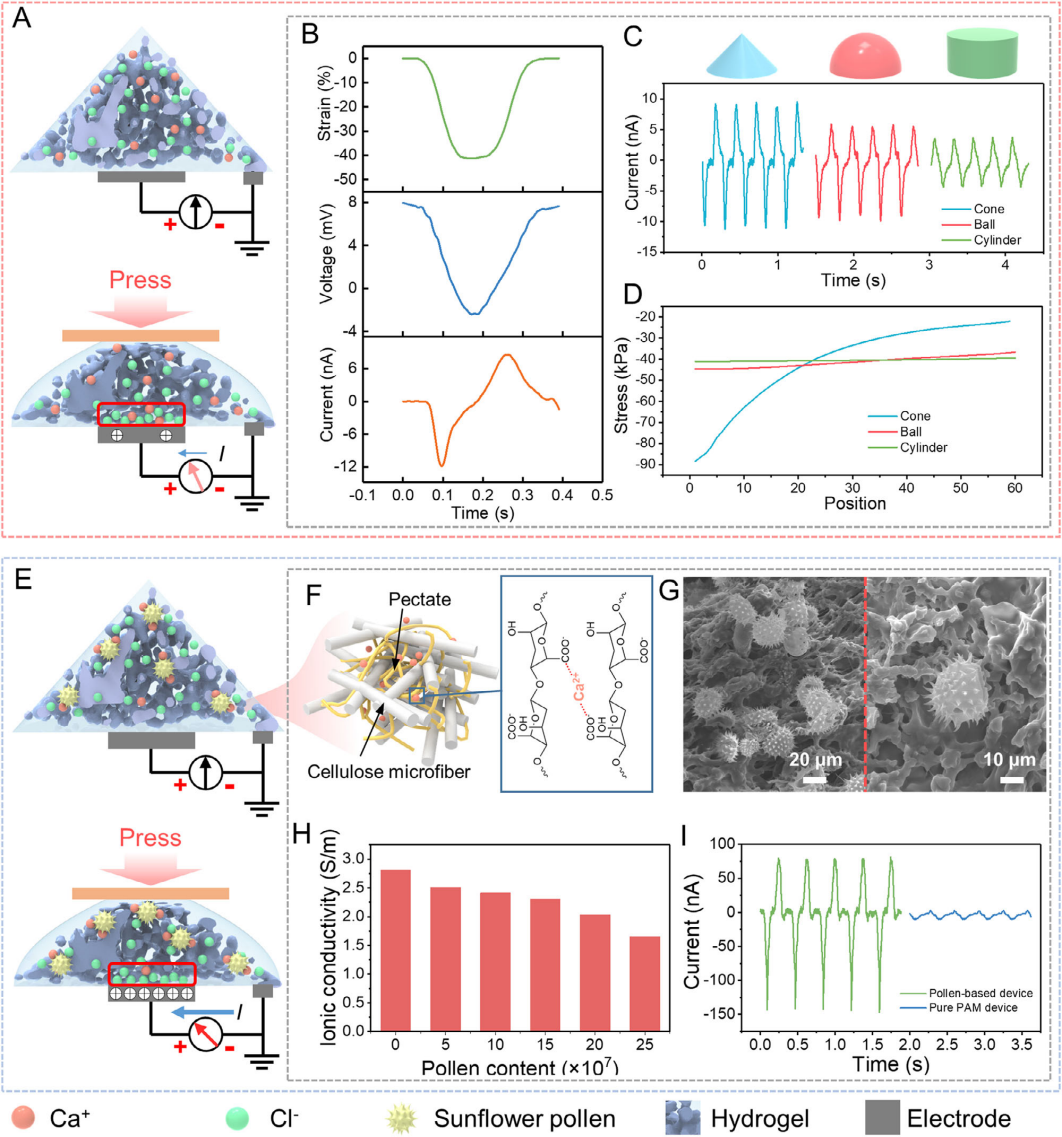
Working principle of PAM‐based bionic mechanoreceptor and output enhancement mechanism of plant‐enhanced bionic mechanoreceptor. (A) The current generation process of PAM‐based mechanoreceptor under pressure. (B) Output electrical signals variation of PAM‐based mechanoreceptor with the press displacement. (C) The current output of PAM‐based mechanoreceptor with different hydrogel geometrical shapes. (D) Simulated stress distribution of different shaped hydrogels along the vertical centerline under the pressed condition. (E) The current generation process of plant‐enhanced mechanoreceptor under pressure. (F) Schematic illustration of calcium ions be captured between pectin molecules. (G) SEM images of freeze‐dried plant‐enhanced PAM hydrogel. (H) Ionic conductivity of plant‐enhanced PAM hydrogel with different pollen content. (I) Comparison of the current output between PAM‐based and plant‐enhanced mechanoreceptor.

To gain deeper insight into the working principle of the plant‐enhanced bionic mechanoreceptor, we first fabricated a PAM‐based bionic mechanoreceptor using pure PAM hydrogel. The ionic signal generation process is demonstrated in Figure 2A and Figure S3. The designed cone‐shaped hydrogel introduces inherent structural asymmetry. Upon compression, a stress gradient will be established along the vertical direction due to the asymmetric deformation induced by the cone geometry (Figure S4a). This gradient drives electrolyte flux from the high‐pressure region at the top toward the low‐pressure region at the bottom through the pore channels within the hydrogel matrix. Furthermore, because the electrolyte is an aqueous CaCl_2_ solution, the hydrodynamic radius of Ca^2^^+^ is larger than that of Cl^−^ due to hydration effects (Figure S15a) [35, 36]. As a result, Ca^2+^ migration is strongly hindered by the hydrogel porous matrix, causing it to be slower than Cl^−^ [37]. This leads to the accumulation of Cl^−^ in the central‐bottom region, creating an ion‐induced potential difference between the center and edges. The resulting potential difference drives a current through the external circuit to balance the internal ion polarization field.

The COMSOL Multiphysics finite element simulation further supports the mechanism of signal generation in hydrogels under compressive deformation. As detailed in Text S1, the simulation integrates poroelastic mechanics modeling with the Poisson–Nernst–Planck (PNP) equations to capture the transient evolution of pore pressure and electric distributions within the hydrogel network (Figure S2a) and electric potential (Figure S2b,c) within the hydrogel during compression. By coupling ionic transport with the deformation of the fluid‐saturated polymer network, the model elucidates the spatiotemporal redistribution of ions and electrochemical gradients that arise from mechanical loading. The results confirm that compression generates a vertical pore pressure gradient in the hydrogel, with elevated pressure at the top and reduced pressure at the bottom, resulting in directional water and ion transport driven by Darcy's law [30]. Due to differences in ion migration rates, faster‐moving anions will accumulate in bottom‐central area, leading to the generation of an electric potential difference between the bottom‐center and peripheral regions. The effective/apparent ion‐transport parameters and other model assumptions used in the poroelastic mechanics and Poisson–Nernst–Planck (PNP)‐based simulation are summarized in Table S1. These parameters were adopted for qualitative illustration of asymmetric ion redistribution in the hydrogel system and should not be interpreted as molecular diffusion coefficients of free ions in bulk aqueous solution.

Mechanical simulations (Figure S4a) indicate that, in addition to the dominant vertical pressure gradient, a secondary lateral pressure gradient develops along the bottom region of the cone. This lateral component can drive partial redistribution of anions from the bottom‐center toward the peripheral areas. However, because the vertical pressure difference imposed by the conical geometry is substantially greater than the lateral contribution, vertical ion transport dominates the overall process. Consequently, the net ionic polarization is primarily governed by vertical transport, leading to an enrichment of anions near the central electrode. The recorded open‐circuit voltage and short‐circuit current from the external circuit (Figure 2B) provide further evidence supporting the proposed working mechanism. The electrode at the center was defined as positive, and the peripheral electrode as negative. Under 50% compressive strain, the device produced a negative voltage and current, consistent with the accumulation of negative charges in the bottom‐central region. Upon release, the voltage returned to baseline, accompanied by a transient positive current. It is worth noting that incomplete electrochemical equilibrium at the electrode–electrolyte interface can lead to a constant background offset in voltage and current—a phenomenon commonly observed in iontronic devices [29, 30, 31]. This constant background signal is analogous to the resting potential of human cells, contributing to the generation of bioinspired ionic feeling signals [29]. From the insight into the working principle, it could be easily found that the geometrical configuration of the hydrogel will significantly affect the output of the bionic mechanoreceptor by shaping the stress gradient during compression. To explore this, we investigated the output of bionic mechanoreceptors fabricated by hydrogel with three different geometry structures, e.g., cone, ball, and cylinder (Figure 2C), and simulated the stress distribution along the central axis of each hydrogel when compressing them to 40% strain (Figure 2D and Figure S4). Compared with ball and cylinder‐structured hydrogels, the cone‐shaped hydrogel exhibited the highest output, attributable to the steep variation of the stress gradient.

Then, we further used a pollen‐based hydrogel to boost the output of the bionic mechanoreceptor. The incorporation of the sunflower pollen particle significantly impedes the movement of Ca^2+^, resulting in a higher concentration of Cl^−^ in the bottom‐central region (Figure 2E and Figure S5). The output induced in the external circuit is therefore significantly boosted. The inhibiting effect of pollen on calcium ions arises from two primary aspects: (i) The relatively large size of sunflower pollen particles will be stuck in the channel of the hydrogel (Figure 2G); (ii) The ability of sunflower pollen particles to trap Ca^2+^ through intermolecular electrostatic attraction (Figure 2F) and spatial confinement (Figure S6). The pectate of pollen is rich in deprotonated carboxyl groups, and Ca^2+^ could be captured between pectate chains due to intermolecular electrostatic attraction [38]. The SEM–EDS elemental mapping (Figure S7) further supports this hypothesis. In the pollen‐based hydrogel, the calcium signal is noticeably concentrated around the pollen microgel domains compared with the surrounding PAM matrix, indicating preferential localization and accumulation of Ca^2^^+^ near the pollen interface. Moreover, when neutral sunflower microgels are immersed in a CaCl_2_ solution, they undergo pronounced shrinkage, and the intine layer acts like a cage, restricting the movement of any unbound Ca^2^^+^ ions within the pollen particles. To further validate the role of sunflower particles in anchoring Ca^2+^, we measured the ionic conductivity of hydrogels with different pollen content while keeping the CaCl_2_ concentration (0.1 M) constant (Figure 2H), and the negative correlation could provide direct evidence to prove the hypothesis.

**FIGURE 3:**
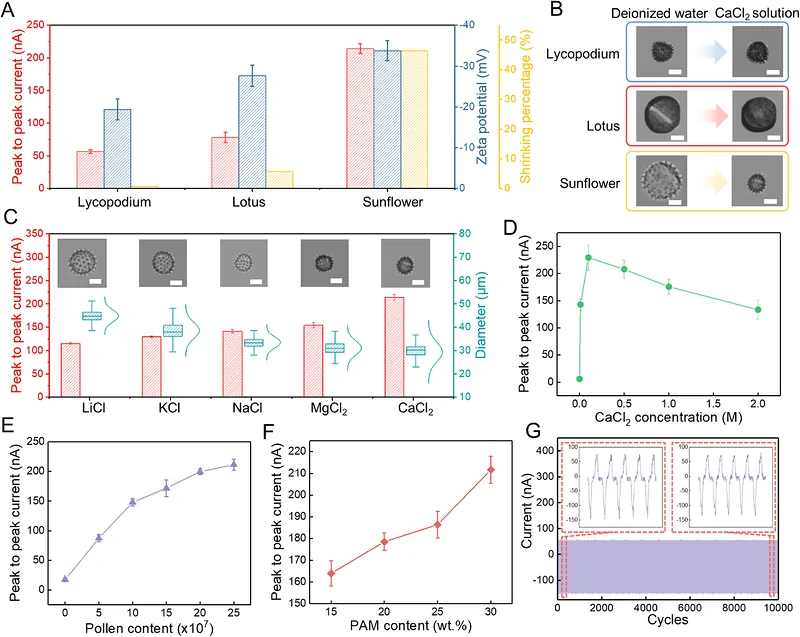
Parameter investigation and optimization of the plant‐enhanced bionic mechanoreceptor. (A) The current output of plant‐enhanced bionic mechanoreceptor fabricated by different pollen species; the Zeta potential of different pollen suspension; shrinking percentage of different pollen species particles in 0.1 M CaCl_2_ solution (original state is in deionized water). (B) Photographs of pollen particles with different species in deionized water and CaCl_2_ solution. (C) The current output of plant‐enhanced bionic mechanoreceptor when using different solutions as the electrolyte; the diameter of sunflower pollen particles in different solutions. The insets are photographs of sunflower pollen particles. (D) The current output variation of the plant‐enhanced bionic mechanoreceptor when increasing CaCl_2_ concentration. (E) The output of plant‐enhanced bionic mechanoreceptor as a function of pollen content. (F) The relationship between current output and PAM content. (G) Current outputs of the plant‐enhanced bionic mechanoreceptor after pressing for 10^5^ cycles. Note: Data are presented as mean ± SD based on five repeated measurements (*n* = 5).

Pollen additives may also reduce ionic conductivity through geometric effects (e.g., increased tortuosity or partial obstruction of ion‐transport pathways). To evaluate the respective roles of geometric effects and pollen‐specific ion interactions, we measured the ionic conductivity of pristine PAM hydrogel and PAM hydrogels containing Lycopodium pollen microgels, Lotus pollen microgels, or Sunflower pollen microgels (all using 0.01 м CaCl_2_ as the electrolyte). As shown in Figure S8, under the same electrolyte condition, the conductivity decreases from 0.34 S m^−1^ for pristine PAM to 0.25 S m^−1^ for the Lycopodium pollen‐based hydrogel, 0.21 S m^−1^ for the Lotus pollen‐based hydrogel, and 0.18 S m^−1^ for the Sunflower pollen‐based hydrogel. As suggested by Figure 3A,B, Lycopodium has a smaller particle size than sunflower but is expected to present fewer negatively charged surface groups (e.g., −COO^−^), leading to weaker Ca^2^^+^ hindrance and thus a higher conductivity. Lotus pollen has a larger particle size than sunflower, yet its surface is also expected to contain fewer ─COO^−^‐type groups; accordingly, its conductivity remains higher than sunflower's despite the larger particle size. Together, these trends suggest that although geometric effects may contribute to the conductivity decrease, they do not solely determine the observed behavior. Instead, the dominant factor governing the conductivity decrease is the density/availability of negatively charged interfacial groups (e.g., ─COO^−^) on pollen microgels that preferentially hinder Ca^2^^+^ transport.

We further performed poroelastic Darcy flow–Poisson–Nernst–Planck (PNP) simulations to analyze asymmetric ion transport in pristine PAM and pollen‐based hydrogels (Figure S9). The simulations confirm that incorporating pollen effectively lowers the mobility ratio *D_+_*/*D_−_*, thereby strengthening ion‐transport asymmetry. This enhanced asymmetry promotes charge separation under compression and ultimately leads to an amplified piezoionic current response.

Hydrogel stiffness may also influence the piezoionic output by altering the pressure developed under a fixed deformation [39]. Therefore, we characterized the mechanical properties of pristine PAM hydrogels and pollen‐based hydrogels. As shown in Figure S10, incorporating soft pollen microgels does not measurably change the stress–strain response of the PAM hydrogel within the relevant deformation range (0%–70% strain), indicating that the hydrogel stiffness remains essentially unchanged after soft pollen microgel addition. Notably, at the strain level used in the electrical measurements (40%), the measured stresses, and thus the nominal pressures under our loading geometry, are nearly identical for pristine PAM and pollen–PAM samples. These results suggest that the enhanced output is not attributable to stiffness‐induced pressure differences, but rather to pollen‐enabled modulation of ion‐transport dynamics (ion anchoring and increased transport asymmetry).

Ultimately, by incorporating sunflower particles, the output of plant‐enhanced bionic mechanoreceptors could be boosted to ∼12 times (143.4 nA) compared to PAM‐based devices (Figure 2I).

### 2.2 Output Performance and Parameter Investigation of Plant‐Enhanced Bionic Mechanoreceptors

In the light of the working principle, key factors influencing the plant‐enhanced bionic mechanoreceptors' performance were investigated to gain further insights into the device. For all tests, the compression strains of bionic mechanoreceptors were fixed at 40%. To begin with, the influence of pollen species on the current generation was studied (Figure 3A). We used three categories of pollen to fabricate bionic mechanoreceptors (e.g., Lycopodium, Lotus, and Sunflower, each with an equal pollen particle content of 2 × 10^8^ in the hydrogel). Among these, the sunflower pollen‐based device has the most excellent output (Figure 3A, red bars), which could also be attributed to intermolecular electrostatic attraction forces and spatial confinement effects as mentioned in the mechanism part. The zeta potential, which reflects the surface charge density of colloidal particles in water, primarily indicates the density of deprotonated carboxyl groups in neutral pollen grains. Notably, sunflower pollen displayed the highest zeta potential (Figure 3A, blue bars), along with the greatest shrinkage percentage upon exposure to CaCl_2_ solution (Figure 3A, yellow bars; Figure 3B and Figure S11), resulting in an exceptionally strong Ca^2^^+^ anchoring effect and superior device performance. The water content of microgels prepared from different pollen species further supports this observation, as higher carboxyl group content promotes water absorption through hydrogen bonding within the hydrogel (Figure S12a). Consistent with the electrical measurements, sunflower microgels exhibited the highest water content (Figure S12b). Additionally, the most stable colloidal state of sunflower microgel, as demonstrated in Figure S12c provides further evidence of its high zeta potential.

Based on the analysis of the mechanism, the ion‐pollen particle interplay also plays a crucial role in determining device performance. It can therefore be explained that the output of sunflower pollen‐based bionic mechanoreceptors varies when using different salt solutions as hydrogel electrolytes (Figure 3C). We could observe that bionic mechanoreceptors containing CaCl_2_ solutions have the largest current output, which can be attributed to the pronounced shrinkage of sunflower pollen particles in the presence of CaCl_2_ (Figure S13), indicating that Ca^2+^ has a stronger interaction with sunflower pollen particles and will be more tightly pinned.

After identifying the optimal pollen species and ion category, we further optimized additional fabrication parameters, including CaCl_2_ concentration and pollen content. When we increased the CaCl_2_ concentration from 0 to 2 м, the current outputs of plant‐enhanced mechanoreceptors initially rose sharply, reaching a maximum at 0.1 м, and then gradually declined (Figure 3D). This phenomenon could be attributed to the trade‐off effect between the quantities of mobile ions and the hydrodynamic radius of Ca^2+^. At lower concentrations, the increasing availability of free ions enhances the current output [29, 30]. However, at higher concentrations, the hydrodynamic radius of Ca^2^^+^ decreases (Figure S14) [40, 41], reducing the migration rate difference between Ca^2^^+^ and Cl^−^. As a result, more Ca^2^^+^ accumulates in the bottom‐central region during compression, diminishing the ion‐induced potential difference and thus lowering the output. Whereas the current output enhancement, on account of the increase in pollen content, is mainly reasoned by the growth number of the overall anchor position provided through the deprotonated carboxyl groups of sunflower pollen (Figure 3E). It should be noted that under the current fabrication conditions, the maximum achievable pollen content was 2.5 × 10^8^, limited by the natural leakage of deionized water from the sunflower pollen microgel. However, employing centrifugation during preparation could potentially further increase the pollen loading.

In addition, the migration rate differential between Ca^2+^ and Cl^−^ also could be rooted from the hindrance effect imposed by the hydrogel matrix, which is further confirmed by the results in Figure 3F and Figure S15. From the SEM images of freeze‐dried hydrogel (Figure S15b), we could observe that when increasing the PAM content, the porous channels inside will become more narrow. That will cause the hydrogel matrix to have a stronger hindering or filtering effect on the ions (Figure S15c) [29], thereby improving the electrical outputs. Eventually, owing to the good encapsulation of the Eco‐flex layer, the current output of the optimized plant‐enhanced bionic mechanoreceptor could exhibit no significant degradation after 10 000 cycles of operation. As a bionic mechanoreceptor, the relationship between the current output and applied force is further evaluated (Figure S16). The piezoionic coefficient (defined as Δ𝑉/Δ𝑃 or Δ𝐼/Δ𝑃) exhibits a distinct two‐stage behavior. For the PAM‐based bionic mechanoreceptor, the coefficient is 2.7 × 10^−^^7^ V Pa^−1^ (3 × 10^−^^4^ nA Pa^−1^) at applied pressures below 22 kPa, and decreases to 7.0 × 10^−^^8^ V Pa^−1^ (1.4 × 10^−^^4^ nA Pa^−1^) within the 22–55 kPa range. In contrast, the plant‐enhanced bionic mechanoreceptor achieves significantly higher values under the same conditions, reaching 1.84 × 10^−^^6^ V Pa^−1^ (6.7 × 10^−^^3^ nA Pa^−1^) below 22 kPa and 4.8 × 10^−^^7^ V Pa^−1^ (1.8 × 10^−^^3^ nA Pa^−1^) in the 22–55 kPa range.

To contextualize these results, we provide a benchmarking comparison with representative piezoionic devices reported in the literature, as summarized in Figure S23 and Table S2.

### 2.3 Feasibility of Sensory‐Enhanced Prostheses

**FIGURE 4:**
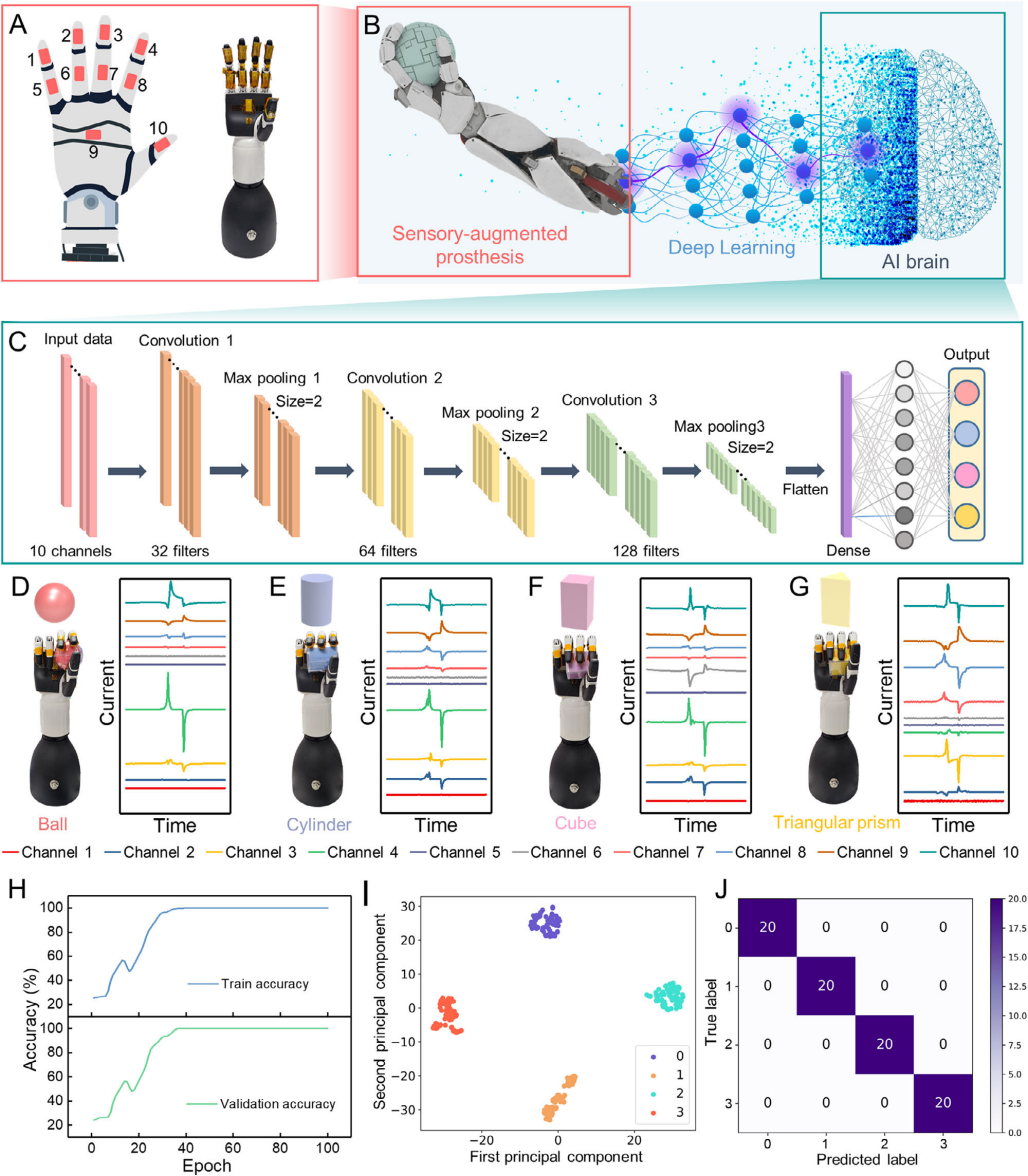
Feasibility prediction of sensory‐enhanced prostheses consisted of plant‐enhanced bionic mechanoreceptors. (A) The distribution of plant‐enhanced bionic mechanoreceptors array on a hand‐shaped prosthesis. (B) The schematic illustration of the simulation process by means of deep learning method to prove the feasibility of sensory‐enhanced prostheses. (C) The structure of a deep learning model. (D‐G) Ionic signals collected from the mechanoreceptor array when controlling the prostheses grab objects with different geometry. (H) The accuracy variation of the training set and validation set during the deep learning process. (I) The visualized clustered results of the dataset. (J) The confusion matrix of deep learning results.

In this work, we proposed a sensory‐enhanced prosthesis concept, where integrating the plant‐enhanced pollen‐based bionic mechanoreceptor into a prosthetic device enables the generation of bioinspired ionic signals during operation, potentially providing the brain with naturalistic neural analogs feedback. Admittedly, directly sending ionic signals to the human brain remains a significant challenge with current technologies. The human brain functions as a deep and complex recurrent neural network [42], and with the booming of artificial intelligence (AI), it is now possible to utilize coding strategiesto construct AI neural network models to reasonably simulate and estimate brain activities [43]. In this context, we further established a system that consists of a sensory‐augmented prosthesis and an AI brain (Figure 4B) to prove the concept. For the prosthetic demonstration, we employed a robotic hand as a prototype and integrated ten plant‐enhanced bionic mechanoreceptors onto its surface to create a sensory‐augmented prosthesis system (Figure 4A). The integrated array exhibits clear spatial discrimination with negligible channel crosstalk (Figure S17). The integrated robotic arm was then programmed to grab four objects with distinct shapes (e.g., ball, cylinder, cube, and triangular prism), while the ionic signals generated by 10 mechanoreceptors were recorded simultaneously by a custom‐designed multichannel acquisition printed circuit board (PCB) (Figure 4D–G, Videos S1–S4, and Figure S19).

Leveraging architectures with multiple hidden layers, deep learning models can represent complex functions more efficiently, making them well‐suited for processing diverse data types and addressing challenging problems. This capability highlights the potential of deep learning in simulating brain activity [43]. Given that the ionic signal is time‐sequential, we first selected a commonly used one‐dimensional (1D) CNN model for the AI brain component. The model consists of a 10‐channel input layer, three convolution layers with 32, 64, and 128 filters, respectively, followed by three max‐pooling layers with a size of 2, and a 1 fully connected layer that outputs 4 object prediction results (Figure 4C).

We defined one complete grab‐and‐release cycle of the robotic arm as one data sample. A dataset comprising 400 samples (each category 100 samples) was established to implement the deep learning task. Besides, each category's dataset was further separated into a training set (60%), a validation set (20%), and a testing set (20%). After 40 epochs of deep training, the convolutional neural network achieved a recognition accuracy of 100% (Figure 4H). Then, we further used the t‐distributed stochastic neighbor embedding (t‐SNE) algorithm to visualize the results, and the data samples from four categories are labeled as “0‐3,” respectively (“0”: Ball, “1”: Cylinder, “2”: Cube, “3”: Triangular prism). The clustered scatter result is shown in Figure 4I, which shows clearly clustered features for four categories. The confusion matrix of deep learning (Figure 4J) could further verify the excellent object recognition capability. Furthermore, we incorporated four additional objects, expanding the dataset to a total of eight categories. As shown in Figure S18, the recognition accuracy remained at 100% even with the enlarged object set. In addition, we utilize the Spiking Neural Networks (SNN) model, a class of artificial neural networks that more closely resemble the way biological brains process information [44], as the AI brain to further prove the feasibility of sensory‐augmented prosthesis. The structure of SNN is illustrated in Figure S20, which consists of an input layer (1790 channels), a hidden layer (128 units), and an output layer (4 channels). The dataset was split into training and testing sets at a 4:1 ratio (320 training samples, 80 testing samples). Only for 10 epochs of deep learning through SNN, the recognition accuracy could reach 100% (Figure S21a), and the confusion map of classified results is shown in Figure S21b. Then, we manually tested the trained SNN model to demonstrate the intuitive working process of the AI brain. First, a random data sample from the cube category (labeled “2”) was selected and sent to the AI brain. Then, the neuronal membrane potential representing label “2” shows the largest value on the output layer of the AI brain (Figure S22a), and the AI brain will generate a spike to indicate that a cube object is being grabbed (Figure S22b).

This result indicates that the AI brain could successfully recognize different objects through the bioinspired ionic signals provided by our bionic mechanoreceptors. This finding further supports the feasibility of using sensory‐augmented prostheses to deliver precise, lifelike tactile feedback, potentially enabling future prosthetic systems to restore a sense comparable to that of natural body parts.

## 3 Conclusions

Here, we developed a planer‐shaped plant‐enhanced bionic mechanoreceptor inspired by the mechanoreceptors in human skin, offering a promising solution for sensory‐augmented prostheses. By leveraging sunflower pollen particles, the ionic polarization within the hydrogel matrix could be significantly enhanced. Under optimized conditions, including selection of pollen species and loading, ion type and concentration, and hydrogel composition, the plant‐enhanced device achieved a current output up to 12 times higher than that of conventional PAM‐based devices. A sensory‐augmented prosthesis was constructed by integrating 10 plant‐enhanced mechanoreceptors into a robotic arm prototype, enabling the collection of bioinspired ionic signals during object manipulation. Subsequently, using an AI‐based neural processing framework built on deep learning models such as Convolutional Neural Networks (CNNs) and Spiking Neural Networks (SNNs), the capability of the sensory‐augmented prosthesis to deliver precise and naturalistic signal feedback was further demonstrated.

This research not only provides a simple and universal method for enhancing ionic polarization‐based devices, which may have a significant influence on soft electronics, biomedical devices, and brain/human–machine interfaces, but also demonstrates the concept of seamless interaction between humans and sensory‐augmented prostheses, which may greatly improve the quality of life for individuals with limb deficiencies.

## 4 Methods

### 4.1 Materials

Raw bee pollen was purchased from Shaanxi GTL Biotech Co., Ltd, Xi'an, Shaanxi, China. KOH, acetone, diethyl ether, acrylamide, N, N‐methylenebisacrylamid, CaCl_2_, LiCl, KCl, NaCl, and MgCl_2_, 2‐Hydroxy‐2‐methylpropiophenone were all purchased from Sigma‐Aldrich. Eco‐flex was obtained from Smooth‐On, Inc.

### 4.2 Defatting of Bee Pollen

To eliminate pollen kitt, natural pollen grains or bee pollen granules were subjected to a sequential defatting process using water, acetone, and diethyl ether.

First, bee pollen (500 g could be Sunflower, Lotus, Lycopodium, etc., species) was dispersed in of deionized water (1 L) at 50°C and stirred at 1000 rpm using a disperse (IKAr) for 2 h. Then, the suspension was filtered through a nylon mesh with 200 µm pores to remove particulate impurities. Excess water was removed via vacuum filtration. The filtered pollen was further refluxed in 1 L of acetone at room temperature while stirring at 1000 rpm for 3 h. Following this, acetone was removed through vacuum filtration, and the pollen was washed 7–8 times with fresh acetone until the filtrate appeared clear. The washed pollen was then transferred to a glass petri dish and dried in a fume hood for 12 h. After acetone defatting, the dried pollen (∼150 g) was dispersed in 1 L of diethyl ether and stirred at 25°C and 1000 rpm for 2 h. This step was repeated for another 2 h with fresh diethyl ether in each cycle. After that, the pollen was washed twice more with diethyl ether and then dispersed in a fresh 1 L batch of the solvent. It was stirred overnight (12 h) at 25°C and 1000 rpm. Finally, diethyl ether was removed by vacuum filtration, and the defatted pollen sample was air‐dried in a fume hood for another 12 h.

### 4.3 Fabrication of Pollen Microgel

Defatted pollen (5 g could be Sunflower, Lotus, Lycopodium, etc., species) was mixed with KOH solution (50 mL, 10 wt.%) in a PTFE round‐bottom flask. The suspension was stirred at 800 rpm and refluxed at 80°C for 2 h, maintaining an approximate pollen‐to‐KOH solution weight ratio of 1:10. Filtration was performed using a custom device equipped with nylon mesh (pore size: 35 µm/30 µm). The mixture was passed through the mesh and washed repeatedly with fresh KOH until the filtrate became clear. The pollen retained on the mesh was set aside for the next processing stage. Fresh KOH solution was prepared (50 mL) and added to the filtered pollen. The mixture was then divided into two tubes, each containing 25 mL. The tubes were placed in an oven set at 80°C for 40 h. After the heat treatment, the suspension was filtered again through nylon mesh (pore size: 35 µm/30 µm) and rinsed thoroughly with deionized water until the pH of the filtrate reached 7.5, as confirmed with pH‐indicator strips. Finally, the pollen microgel was collected by naturally leaking the deionized water for 2 h, and the final product was stored at 4°C for subsequent use.

### 4.4 Fabrication of Plant‐Enhanced Bionic Mechanoreceptor

The fabrication of plant‐enhanced bionic mechanoreceptor could be divided into three parts (Figure S1a–c).

(i) Bottom silver electrode pattern (Figure S1a): First, a polyimide (PI) film was selected as the substrate and washed with ethanol to remove the contaminants on the surface. Then, the silver electrode patterns were printed by Direct Ink Writing (DIW) equipment (Nordson E3), and the line speed of the injector was set to 10 mm/s. A stretchable silver electronic paste (DM‐SIP‐3060S, DYCOTEC MATERIALS Ltd.) was used during the process. (ii) Cone‐shaped plant‐enhanced hydrogel (Figure S1b): First, 3 g acrylamide (AM) powder, 0.7 g CaCl_2_ (∼1 _M_), 0.02 g N, N‐methylenebisacrylamid was dissolved into 7 g pollen microgel. Then, add 0.06 g 2‐Hydroxy‐2‐methylpropiophenone (2 wt.% vs. AM) into the mixed solution above as the photoinitiator, and string to fully dissolve. Finally, transferring 16 µL above solution into a 3D‐printed cone‐shaped model and exposing it to UV light for 2 min. The pollen microgel could be replaced by DI water to fabricate a PAM‐based bionic mechanoreceptor. CaCl_2_ could be changed to LiCl, KCl, NaCl, or MgCl_2_ for ion category investigation. The content of CaCl_2_, pollen, and AM could also be varied for other parameter investigations. (iii) Eco‐flex encapsulation layer (Figure S1c): 1 g part A and 1 g part B of eco‐flex were mixed together and stirred thoroughly to form a homogeneous mixture. Then, pour the liquid Eco‐Flex into a 3D‐printed model and cure under 50°C for 4 h.

### 4.5 Characterization and Measurements

A step motor (LM1225QI, DAZEJIDIAN Ltd.) was used to input the mechanical stimuli. The electrical output was measured by a multifunction DAQ device (USB‐6211, National Instruments) and a Low Noise Current Preamplifier (SR 570, Stanford Research Systems). SEM was obtained through JSM‐7600F. The diameter measurement of pollen particles was conducted by a Fluid Imaging FlowCAM system (Fluid Imaging Technologies, Scarborough, ME, USA). The ionic conductivity was tested by an electrochemical workstation (AutoLab).

### 4.6 Statistical Analysis

No data preprocessing was performed unless otherwise stated. Quantitative data are presented as mean ± standard deviation (SD) based on five repeated measurements (*n* = 5). No statistical hypothesis testing was performed in this work. Data analysis and plotting were performed using Origin 2017 and Python.

## Conflicts of Interest

NJC and MHK have the technology disclosures related to this manuscript technologics.

## Supporting information




**Supporting File 1**: advs75946‐sup‐0001‐SuppMat.docx.

**Supporting File 2**: advs75946‐sup‐0002‐VideoS1.mp4.

**Supporting File 3**: advs75946‐sup‐0003‐VideoS2.mp4.

**Supporting File 4**: advs75946‐sup‐0004‐VideoS3.mp4.

**Supporting File 5**: advs75946‐sup‐0005‐VideoS4.mp4.

## Data Availability

All data needed to evaluate the conclusions in this paper are present in the paper or the Supplementary Materials.
