# Supplementary material for: A Pollen‐Enhanced Bionic Mechanoreceptor Induced by Asymmetric Ionic Convection in Hydrogel for Sensory‐Augmented Prostheses
Source: Adv Sci (Weinh). 2026 Jun 19:e21235. Online ahead of print. doi: 10.1002/advs.202521235 (PMC13336820; doi:10.1002/advs.202521235)
Supplement: Supplementary file 1 — Supporting File 1: advs75946‐sup‐0001‐SuppMat.docx. [file ADVS-9999-e21235-s004.docx]

Supporting Information

A Pollen-enhanced Bionic Mechanoreceptor Induced by Asymmetric Ionic Convection in Hydrogel for Sensory-augmented Prostheses

Zi Hao Guo, Jingyu Deng, Yanzhang Xu, Chenchen Zhou, Yangshi Shao, Xiong Pu, Munho Kim*, Namjoon Cho*

Dr. Z. H. Guo, Prof. M. Kim

School of Electrical and Electronic Engineering, Nanyang Technological University, 50 Nanyang Avenue, 639798, Singapore.
E-mail: munho.kim@ntu.edu.sg

J. Deng, Y. Xu, Dr. C. Zhou, Prof. N. Cho
School of Materials Science and Engineering, Nanyang Technological University, 50 Nanyang Avenue, 639798, Singapore

E-mail: [njcho@ntu.edu.sg](mailto:njcho@ntu.edu.sg)

J. Deng, Y. Xu, Dr. C. Zhou, Prof. N. Cho
Centre for Cross Economy, Nanyang Technological University, 60 Nanyang Drive, Singapore 637551.

Y. Shao, Prof. X. Pu
Beijing Institute of Nanoenergy and Nanosystems, Chinese Academy of Sciences, Beijing 100083, P. R. China.

Keywords: mechanoreceptors, ionic devices, plant-based, bioinspired, pollen, bionic, prosthesis

**Video S1.** Grasp–release of a ball object and corresponding current signals from the integrated bionic mechanoreceptors.

**Video S2.** Grasp–release of a cube object and corresponding current signals from the integrated bionic mechanoreceptors.

**Video S3.** Grasp–release of a cylinder object and corresponding current signals from the integrated bionic mechanoreceptors.

**Video S4.** Grasp–release of a triangular prism object and corresponding current signals from the integrated bionic mechanoreceptors.

**Supplementary Text 1**

A simulation study was conducted using COMSOL Multiphysics (v6.2) to investigate the electric field distribution in CaCl₂-containing hydrogels subjected to compressive loading at the apex of a conical structure. The study further examined the effect of hydrogel modification with pollen as a variable. Fixed constraints were applied at the base of the cone to evaluate electric field responses under different conditions. The theory models were based on the established piezoionic modeling approach reported in previous paper [30, 31]. The equations listed in the Supplementary are implemented using the corresponding physics interfaces in COMSOL Multiphysics, and the explicit forms and definitions are consistent with the COMSOL official documentation.

In the simulation, the fluid phase was modeled as a water solution which comprising an elastic polymer matrix saturated with a solvent. This configuration enabled fluid convection driven by a pressure gradient, which persisted until counteracted by the expansion of the polymer matrix. The poroelasticity interface was utilized to couple Solid Mechanics with Darcy's Law, establishing the primary governing equations for the system.

The mechanical equilibrium in the solid framework is given by:

$\text{0=}\text{∇⋅}\left( \text{s-}\text{α}_{\text{B}}\left( \text{p}_{\text{A}}\text{-}\text{p}_{\text{ref}} \right)\text{i} \right)\text{+}\text{F}_{\text{V}}$ (1)

where σ is the effective stress tensor, α_B_ is the Biot coefficient, p_A_ is the pore pressure, p_ref_ is the reference pressure, I is the identity matrix, and F_V_ represents volumetric forces like gravity.

Fluid mass conservation is expressed by:

$\frac{\text{∂}\text{ρ}_{\text{f}}}{\text{∂t}}\text{+}\text{∇⋅}\left( \text{ρ}_{\text{f}}\text{v}_{\text{d}} \right)\text{=-}\text{ρ}_{\text{f}}\text{α}_{\text{B}}\frac{\text{∂}\text{ϵ}_{\text{vol}}}{\text{∂t}}\text{+}\text{Q}_{\text{m}}$ (2)

where *ρ_f_* is fluid density, *v_d_* is Darcy velocity, *ε_vol_* is volumetric strain, and *Q_m_* represents mass sources or sinks.

The coupling between fluid pressure and volumetric strain is described as:

$\text{S}_{\text{p}}\text{=}\text{ϵ}_{\text{f}}\text{χ}_{\text{f}}\text{+}\left( \text{α}_{\text{B}}\text{-}\text{ϵ}_{\text{f}} \right)\text{χ}_{\text{s}}$ (3)

where *ε_f_* is the porosity, *χ_f_* is fluid compressibility, *α_B_* is the Biot coefficient, and *χ_s_* is solid compressibility. The solid matrix compressibility is defined as:

$\text{χ}_{\text{s}}\text{=}\frac{\text{1-}\text{α}_{\text{B}}}{\text{K}_{\text{d}}}$ (4)

where *K_d_* is the drained bulk modulus of the solid matrix.

In the simulation, the fluid phase was defined as a water solution comprising an elastic polymer matrix infused with a solvent. This setup enabled fluid convection induced by a pressure gradient, continuing until equilibrium was achieved through polymer expansion. The poroelasticity interface linked Solid Mechanics with Darcy's Law, forming the fundamental governing equations.

The flow velocity output was used to compute transferred mass and charge via the Nernst-Planck equation, substituting for Darcy velocity:

$\frac{\text{∂}\text{c}_{\text{i}}}{\text{∂t}}\text{+}\text{∇⋅}\left( \text{-}\text{D}_{\text{i}}\text{∇}\text{c}_{\text{i}}\text{-}\text{n}_{\text{i}}\text{μ}_{\text{m}}\text{f}_{\text{c}}\text{c}_{\text{i}}\text{∇}\text{V} \right)\text{+}\text{v}_{\text{d}}\text{⋅∇}\text{c}_{\text{i}}\text{=}\text{R}_{\text{i}}$ (5)

where *∂c_i_/∂t* is the rate of change of concentration of substance i over time, *-D_i_∇c_i_* represents diffusion, *-n_i_μmf_c_c_i_∇V* describes electromigration, and *R_i_* is the source or sink term.

The electrostatic potential, accounting for ionic concentration gradients, is determined using Poisson's equation:

$\text{∇⋅}\left( \text{ϵ}\text{∇}\text{ϕ} \right)\text{=-}\sum_{\text{i}} \text{n}_{\text{i}}\text{e}\text{c}_{\text{i}}$ (6)

where *ϕ* is the electric potential, *n_i_* is the charge number, *c_i_* is ion concentration, e is the elementary charge, and *ε* is the medium's permittivity. A distant air-filled sphere is used as the grounding point, with its potential integrated into the Nernst–Planck equation to model the transient redistribution of ions. This coupling enables the calculation of the potential difference generated within the hydrogel when subjected to external forces.

| Parameter name | Value | Unit | Description |
| --- | --- | --- | --- |
| Porosity | 0.75 | 1 | Assumed porosity of the hydrogel network used in the poroelastic model |
| Fluid density | 1.05 | g/cm^3^ | Density of the aqueous electrolyte phase in the hydrogel |
| Permeability | 3×10^-15^ | m^2^ | Hydraulic permeability of the hydrogel network |
| Dynamic viscosity | 1×10^-3^ | Pa·s | Dynamic viscosity of the aqueous phase |
| Effective cation transport coefficient | 1×10^-8^ | m^2^/s | Effective coefficient used to describe cation transport in the qualitative continuum PNP model |
| Effective anion transport coefficient | 1×10^-6^ | m^2^/s | Effective coefficient used to describe anion transport in the qualitative continuum PNP model |
| Relative permittivity | 82.9 | 1 | Relative permittivity of the aqueous electrolyte phase |
| Poisson's ratio | 0.35 | 1 | Assumed Poisson’s ratio of the hydrogel matrix |
| Initial Ca^2+^ concentration | 0.1 | M | Same as the electrolyte concentration used in the experiment |
| Initial Cl^-^ concentration | 0.2 | M | Same as the electrolyte concentration used in the experiment |
| Young’s modulus | 10 | MPa | Elastic modulus of the hydrogel used in the mechanical/poroelastic model |
| Effective polymer weight fraction | 30 | Wt.% | Estimated from the mass of monomer and crosslinker relative to the total precursor mass |

**Table S1.** **Simulation parameter.**

Note: Effective transport coefficient of ionic species are adopted from literature and chosen to be consistent with recent piezoionic modeling reports (e.g., Wang et al., Adv. Mater. 36, e2405391 (2024)). These values are not intended to represent the molecular diffusion coefficients of free ions in bulk aqueous solution, but are used to reproduce the qualitative direction of ion redistribution and the sign of the generated potential.


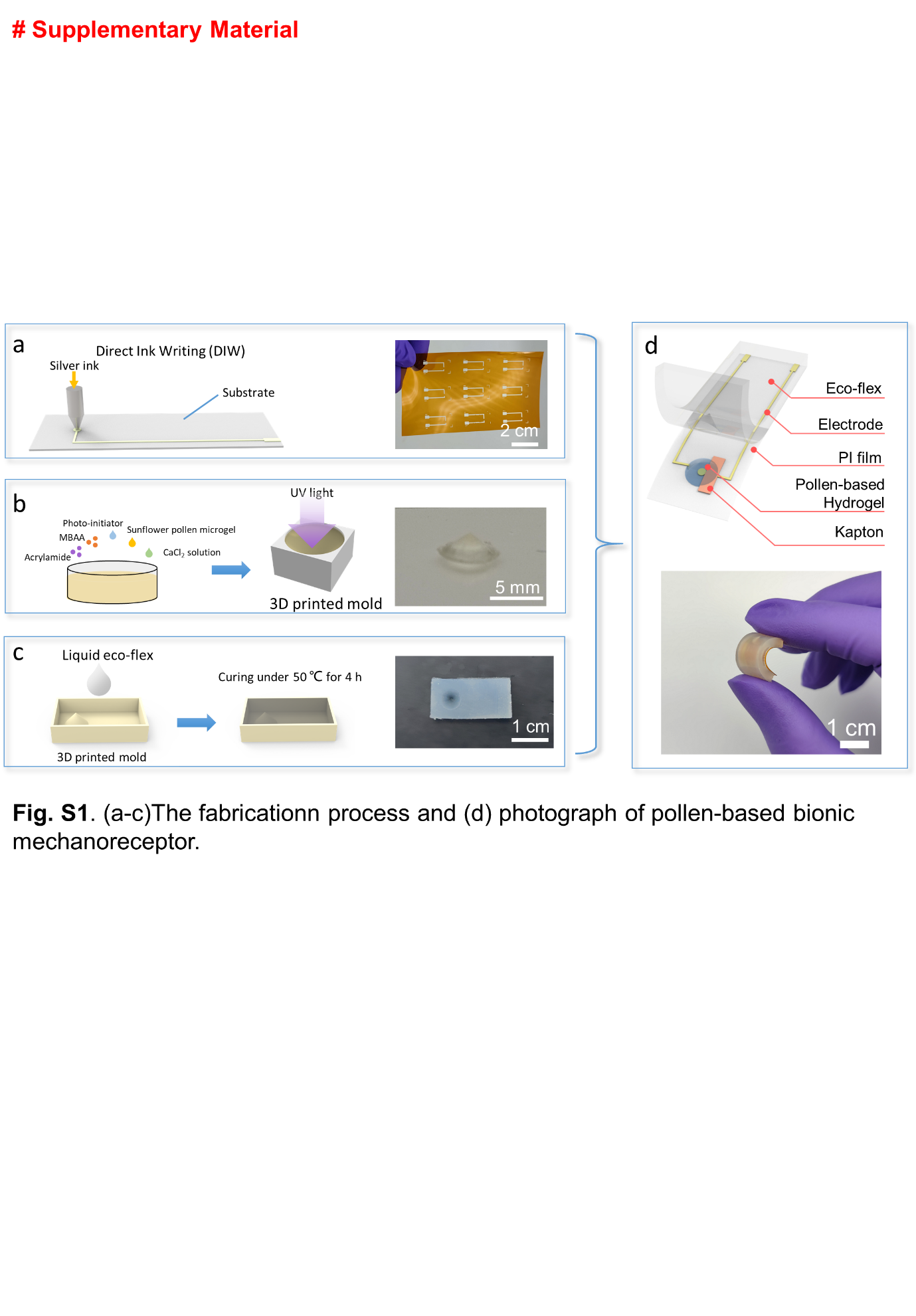


**Figure S1.**

(a-c) The fabricationn process and (d) photograph of pollen-based bionic mechanoreceptor.


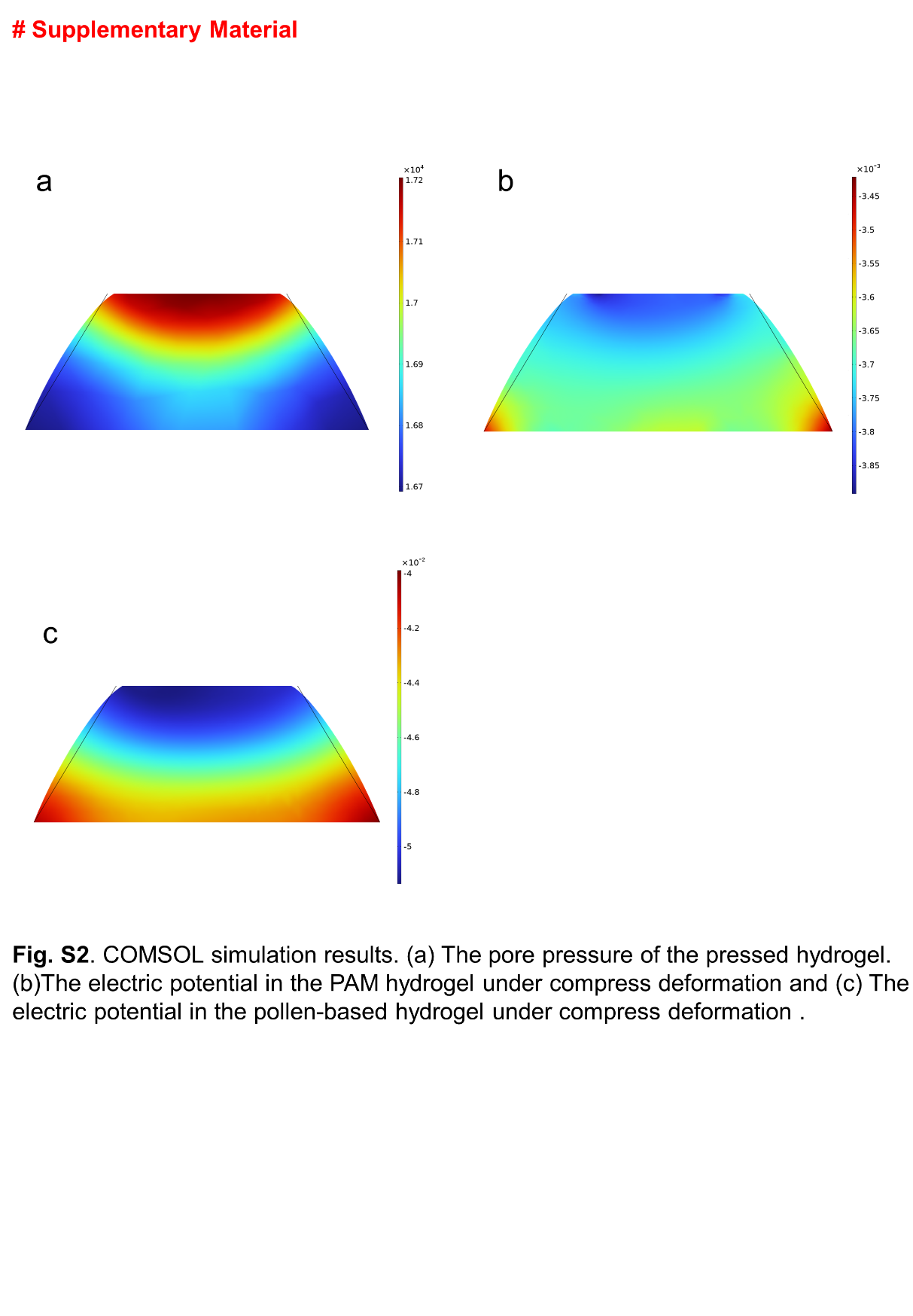


**Figure S2.**

COMSOL simulation results. **(a)** The pore pressure of the pressed hydrogel. **(b)**The electric potential in the PAM hydrogel under compress deformation and **(c)** The electric potential in the pollen-based hydrogel under compress deformation.


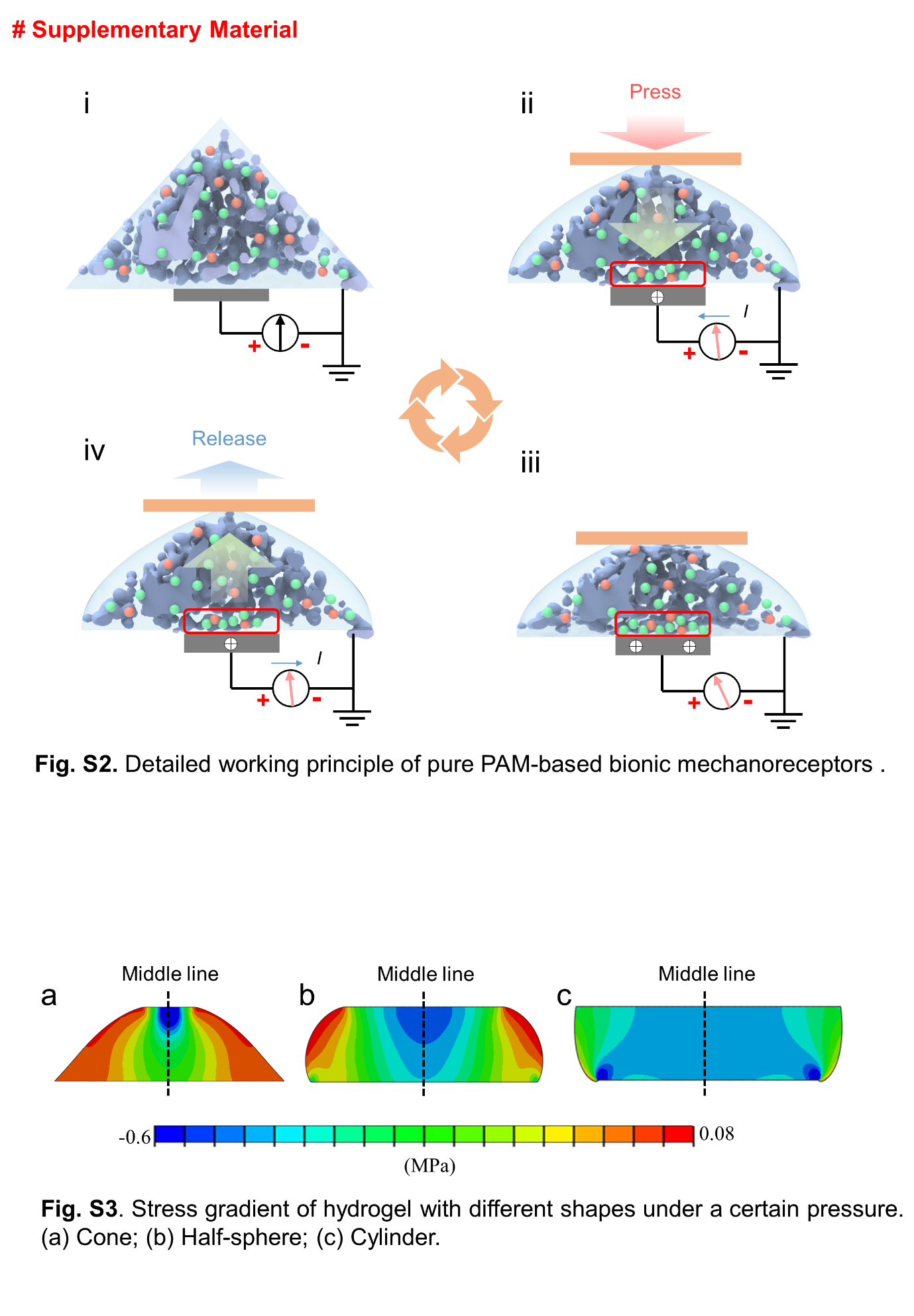


**Figure S3.**

Detailed working principle of pure PAM-based bionic mechanoreceptors.


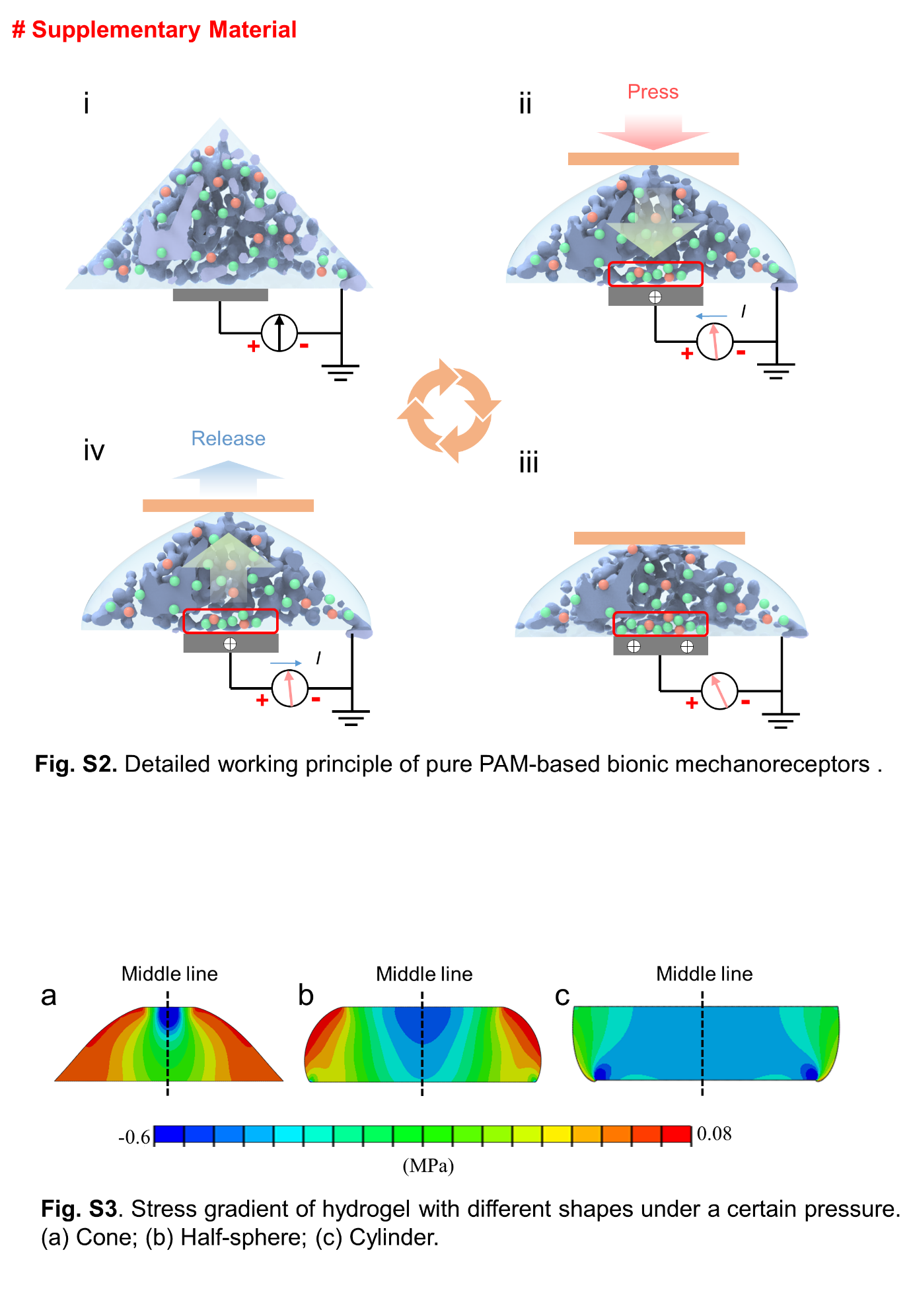


**Figure S4.**

Stress gradient of hydrogel with different shapes under a certain pressure. **(a)** Cone; **(b)** Half-sphere; **(c)** Cylinder.


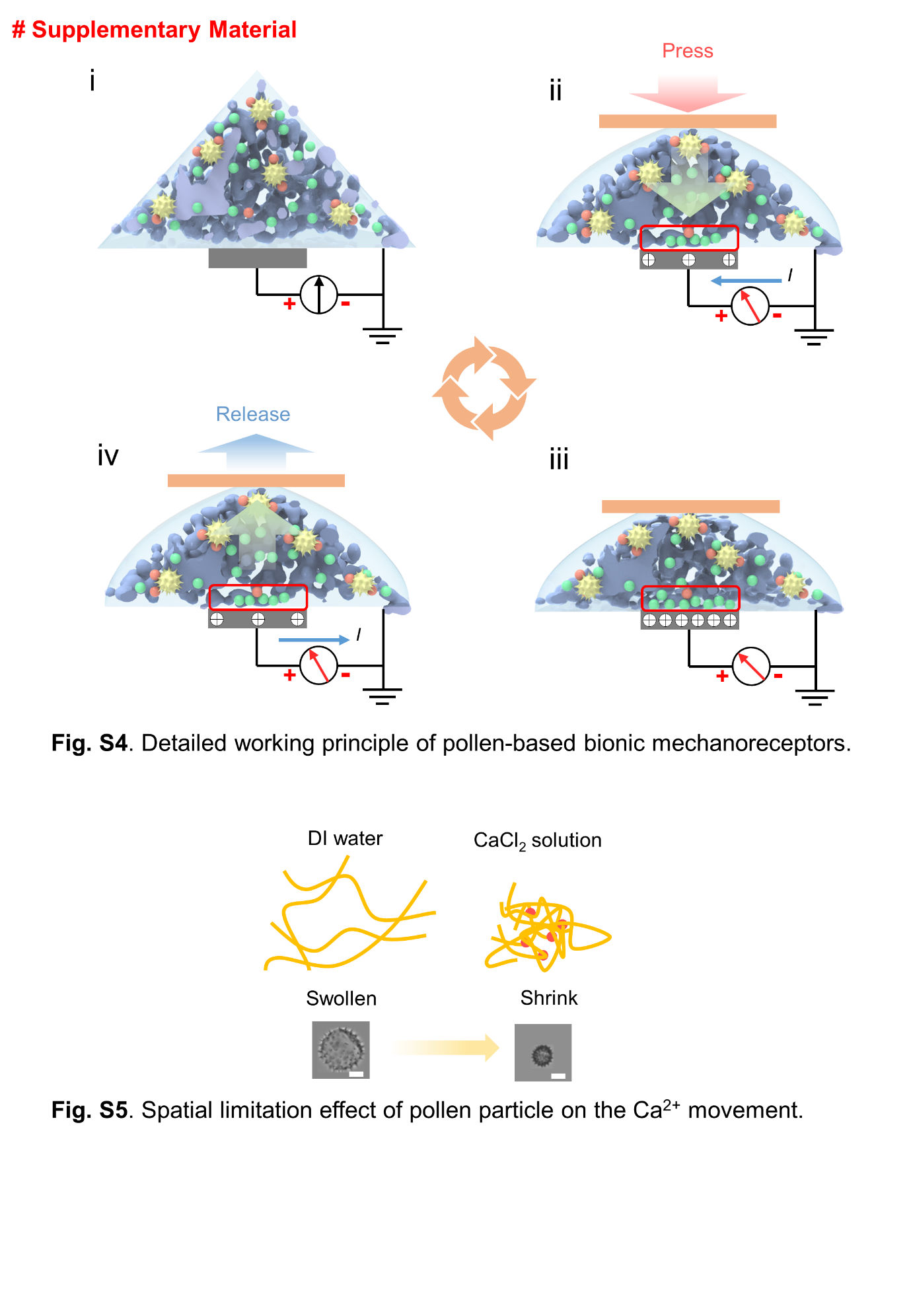


**Figure S5.**

Detailed working principle of pollen-based bionic mechanoreceptors.


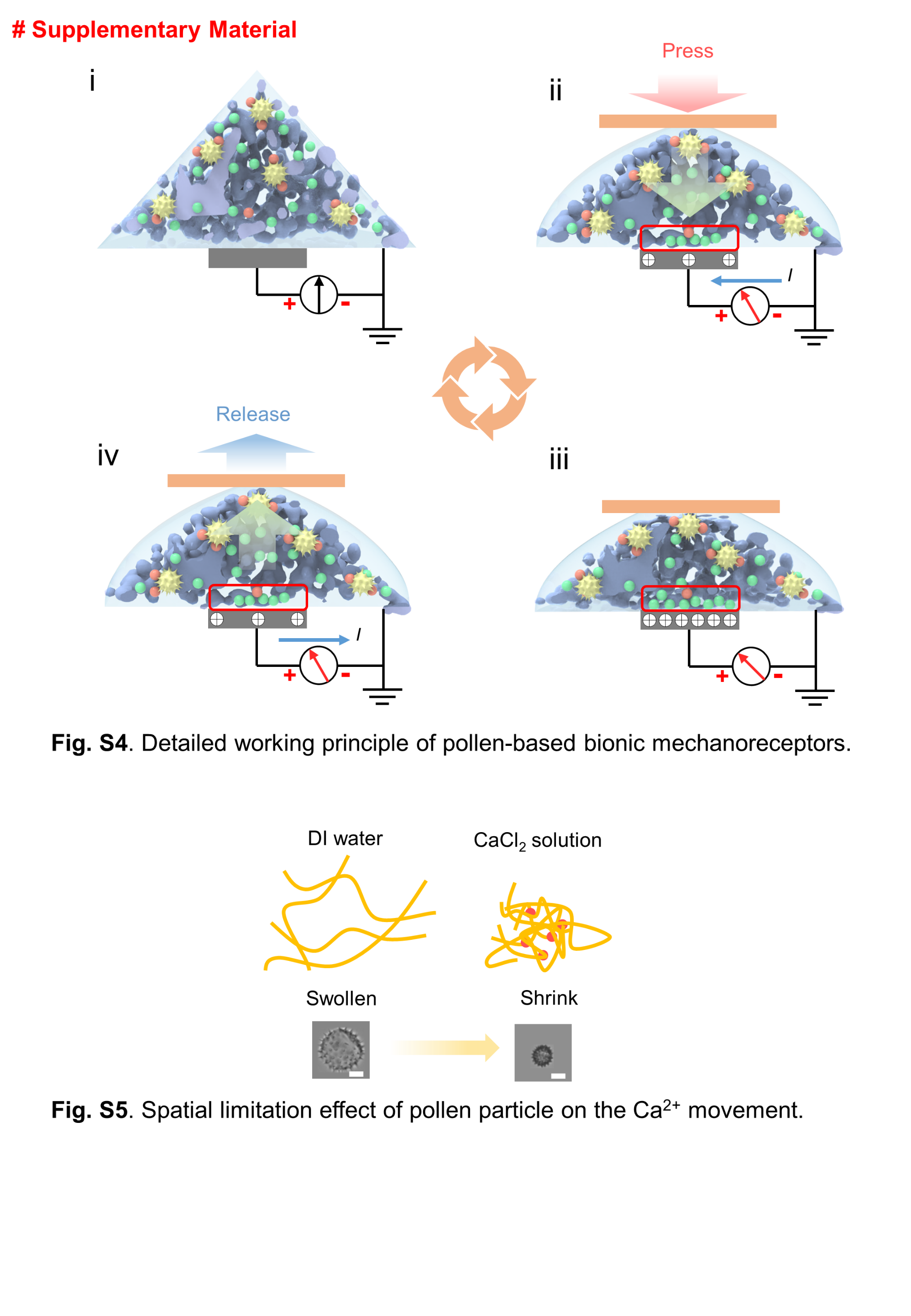


**Figure S6.**

Spatial limitation effect of pollen particle on the Ca^2+^ movement.


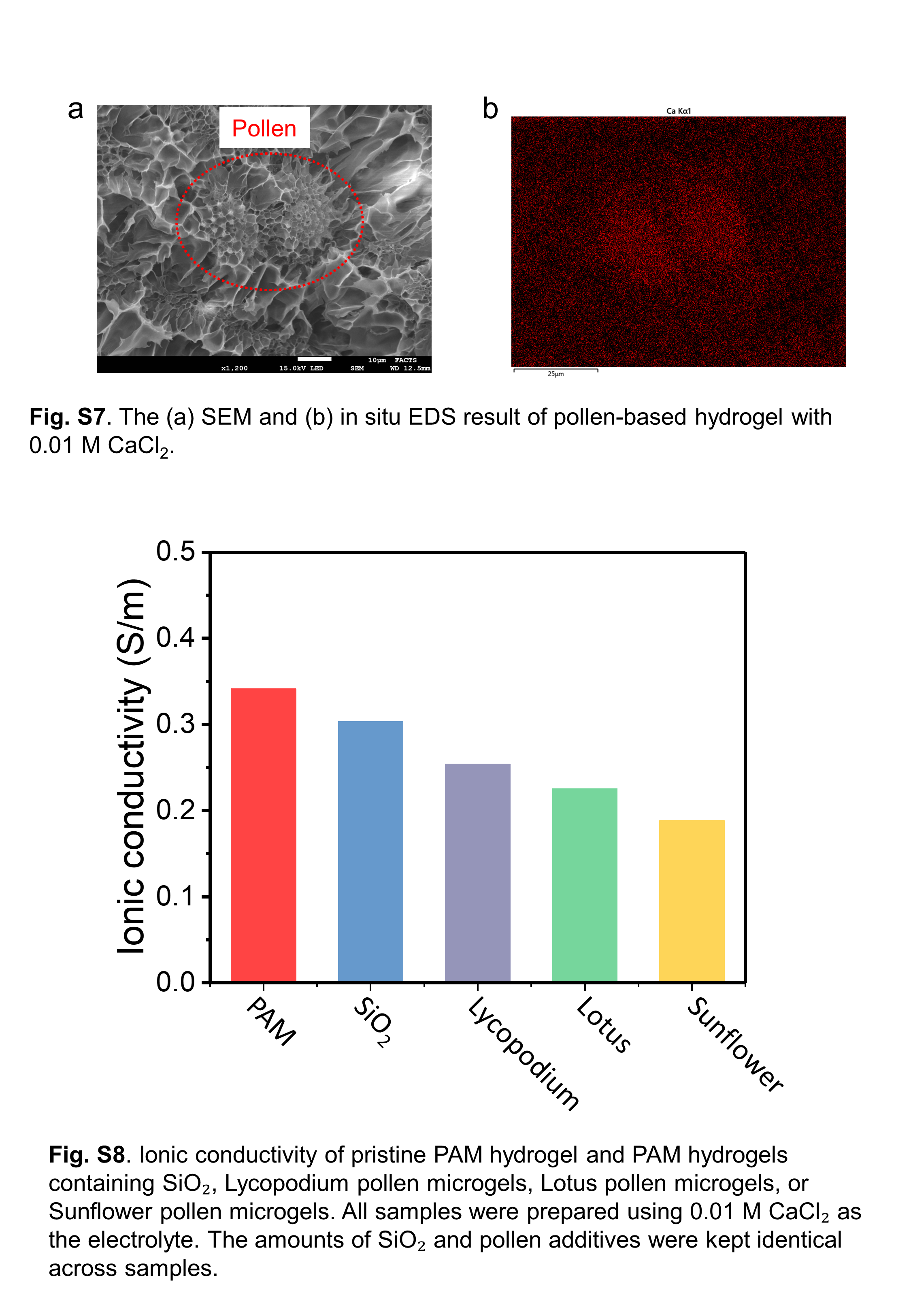


**Fig. S7**. The (a) SEM and (b) in situ EDS result of pollen-based hydrogel with 0.01 M CaCl_2_.


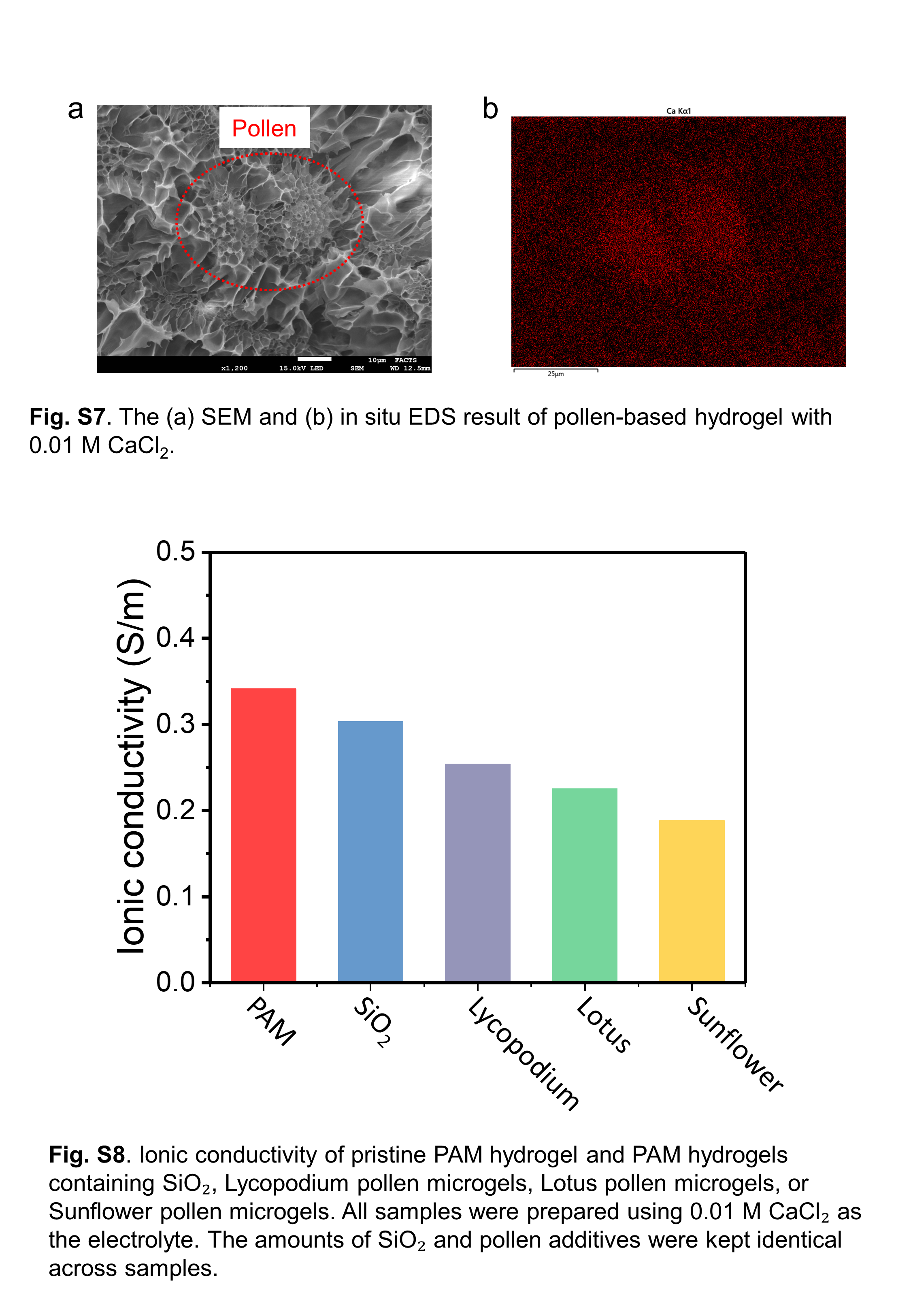


**Fig. S8**. Ionic conductivity of pristine PAM hydrogel and PAM hydrogels containing SiO₂, Lycopodium pollen microgels, Lotus pollen microgels, or Sunflower pollen microgels. All samples were prepared using 0.01 M CaCl₂ as the electrolyte. The amounts of SiO₂ and pollen additives were kept identical across samples.


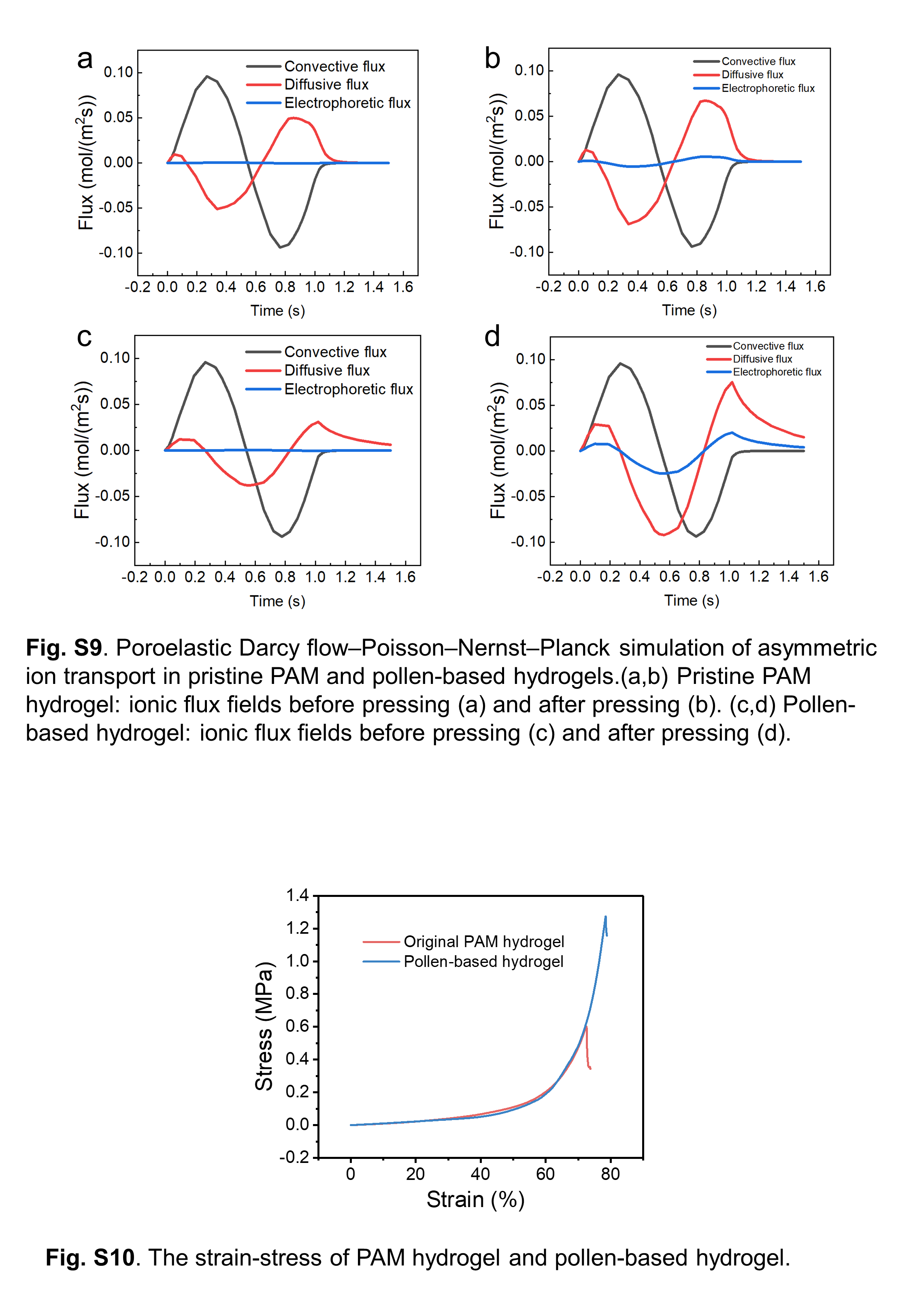


**Fig. S9**. Poroelastic Darcy flow–Poisson–Nernst–Planck simulation of asymmetric ion transport in pristine PAM and pollen-based hydrogels.(a,b) Pristine PAM hydrogel: ionic flux fields before pressing (a) and after pressing (b). (c,d) Pollen-based hydrogel: ionic flux fields before pressing (c) and after pressing (d).


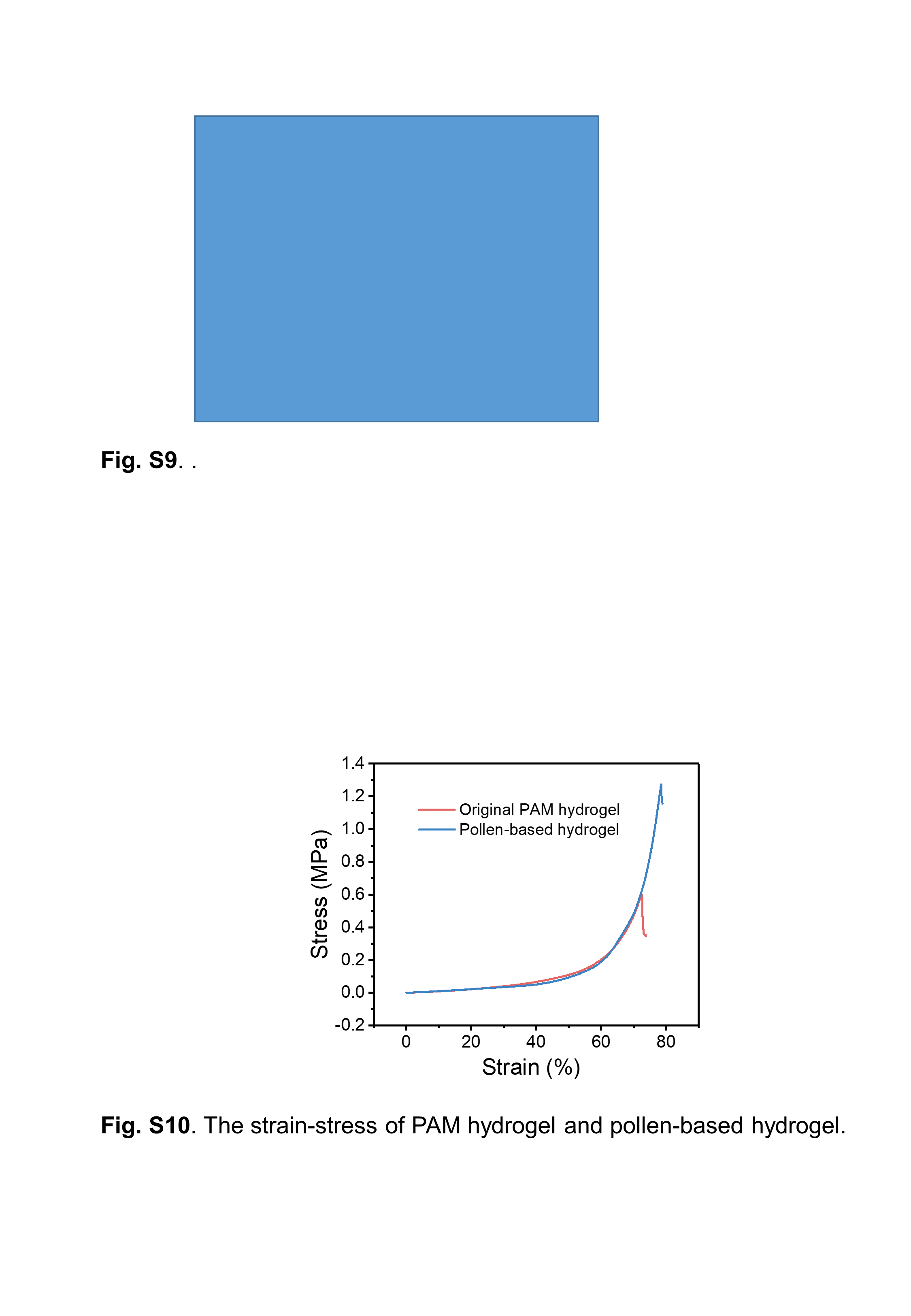


**Fig. S10**. The strain-stress of PAM hydrogel and pollen-based hydrogel.


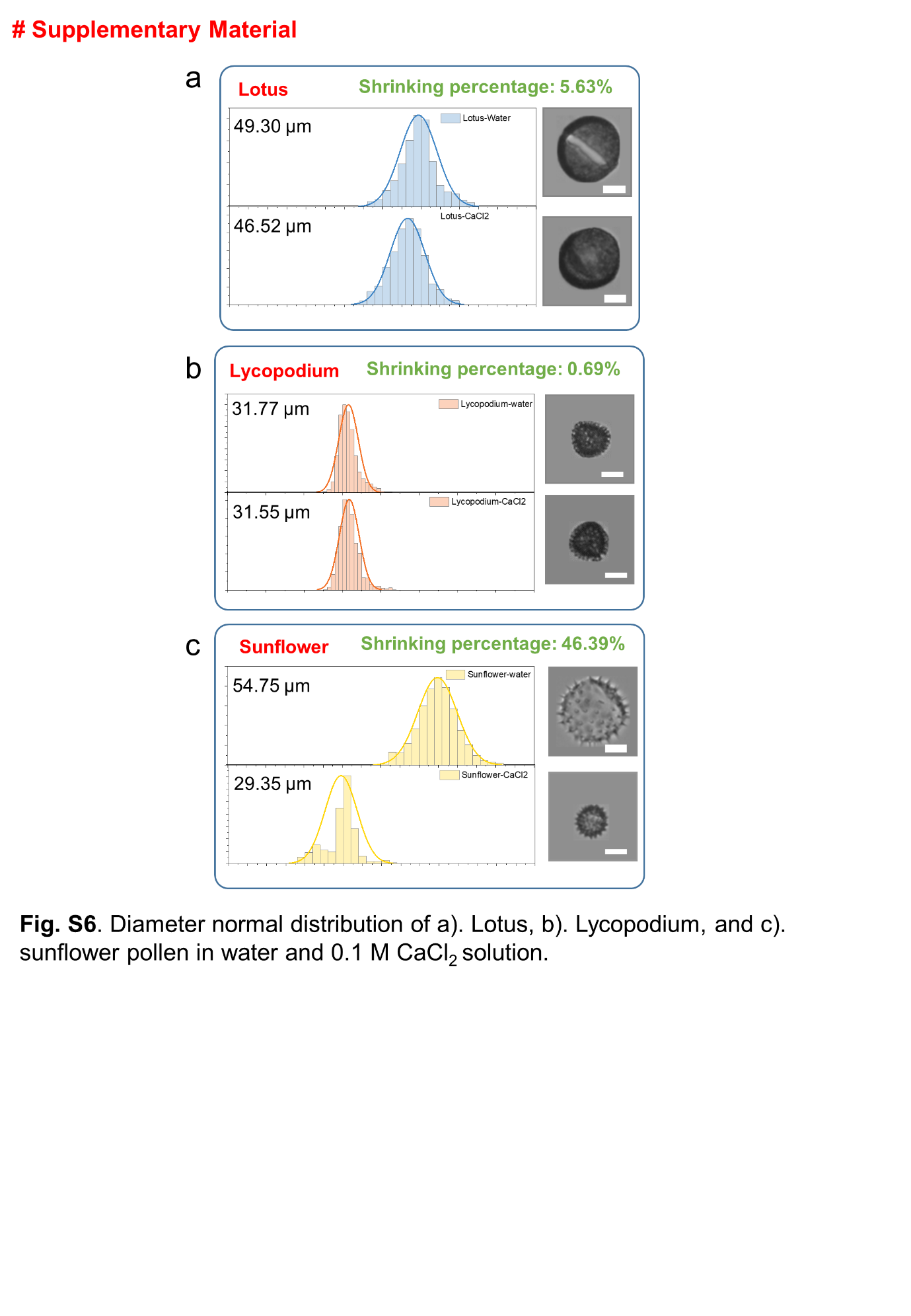


**Figure S11.**

Diameter normal distribution of **(a).** Lotus, **(b)**. Lycopodium, and **(c)**. sunflower pollen in water and 0.1 M CaCl_2_ solution.


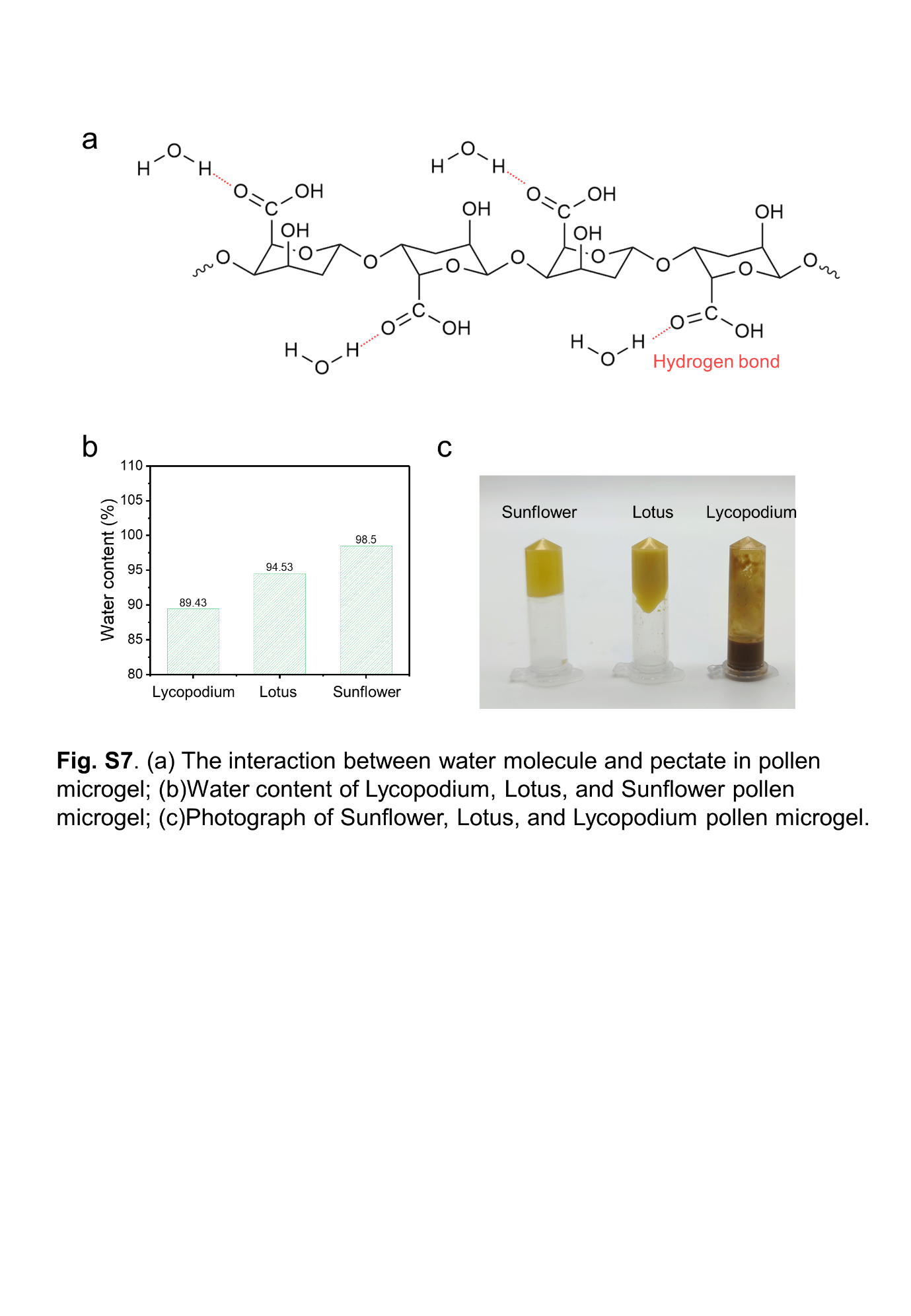


**Figure S12.**

**(a)** The interaction between water molecule and pectate in pollen microgel; **(b)**Water content of Lycopodium, Lotus, and Sunflower pollen microgel; **(c)**Photograph of Sunflower, Lotus, and Lycopodium pollen microgel.


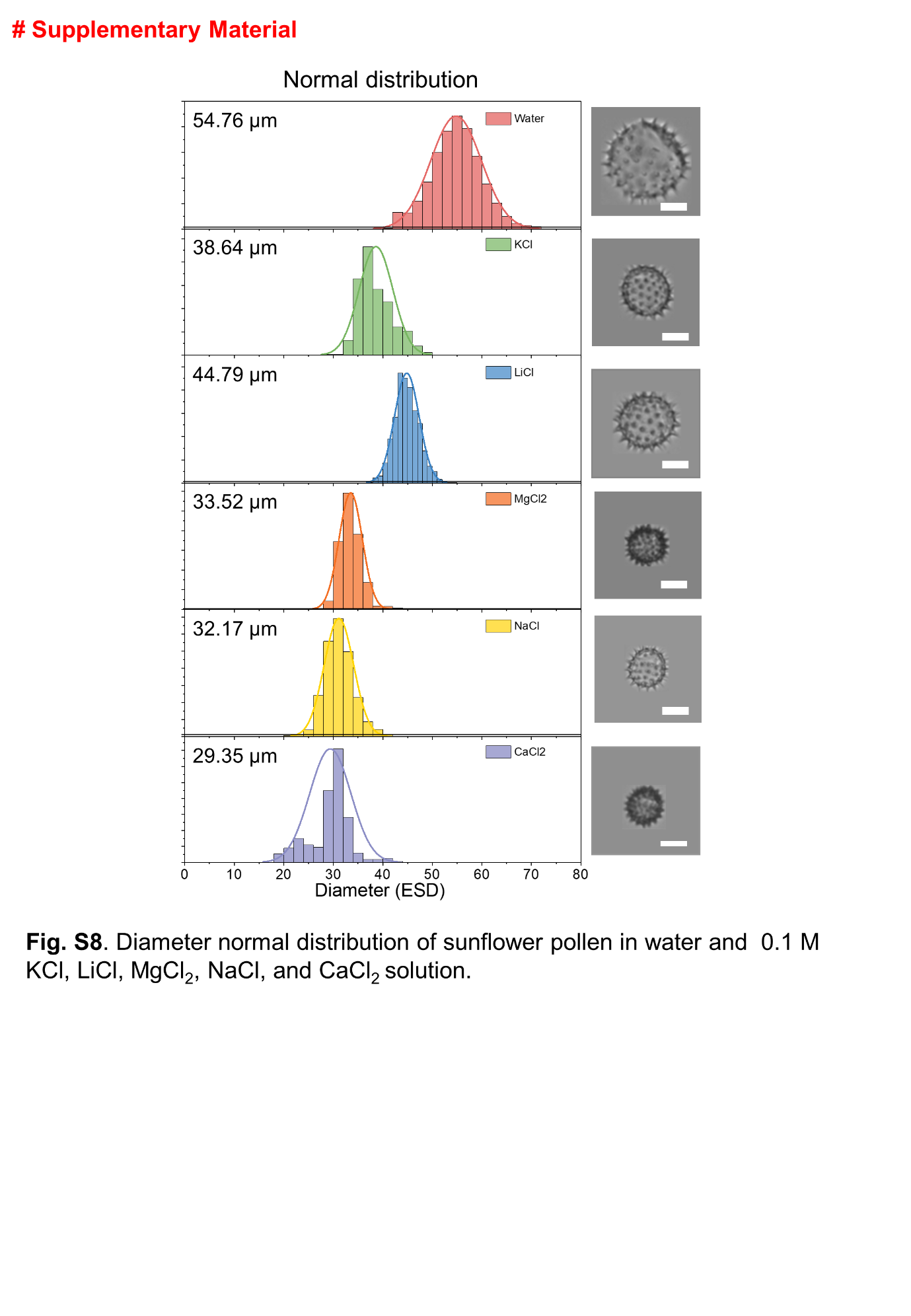


**Figure S13.**

Diameter normal distribution of sunflower pollen in water and 0.1 M KCl, LiCl, MgCl_2_, NaCl, and CaCl_2_ solution.


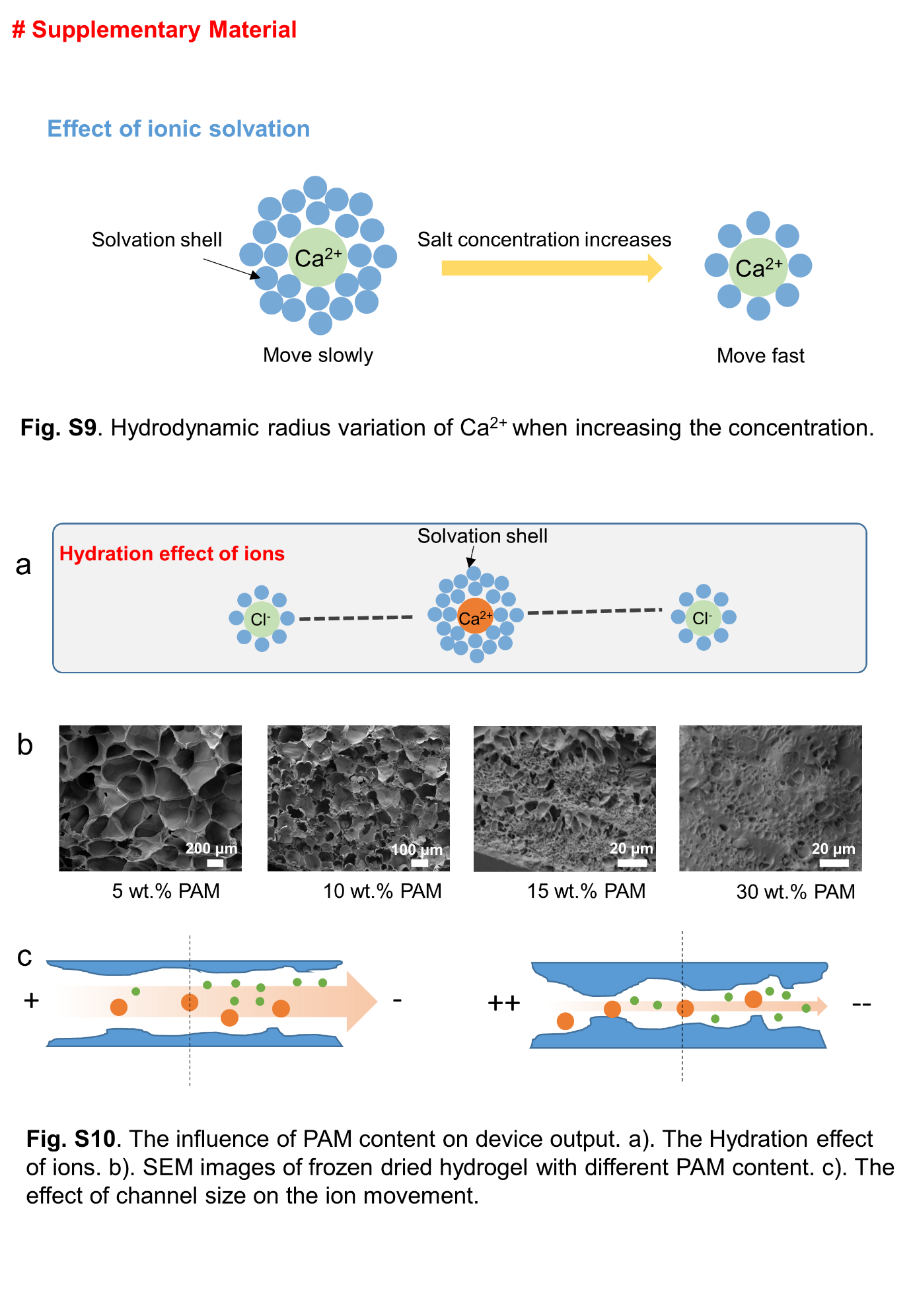


**Figure S14.**

Hydrodynamic radius variation of Ca^2+^ when increasing the concentration.

**
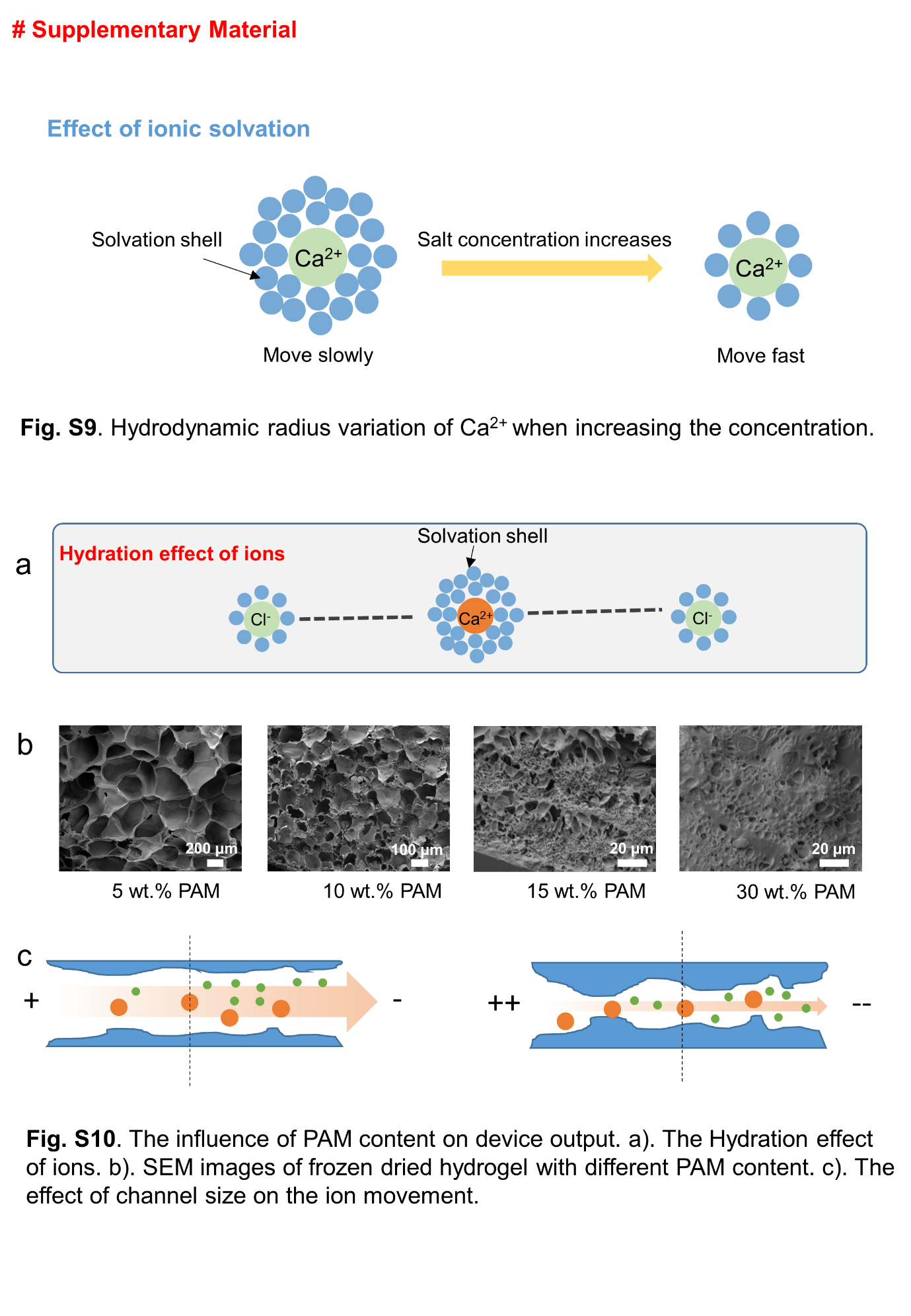
**

**Figure S15.**

The influence of PAM content on device output. **(a)**. The Hydration effect of ions. **(b)**. SEM images of frozen dried hydrogel with different PAM content. **(c)**. The effect of channel size on the ion movement.


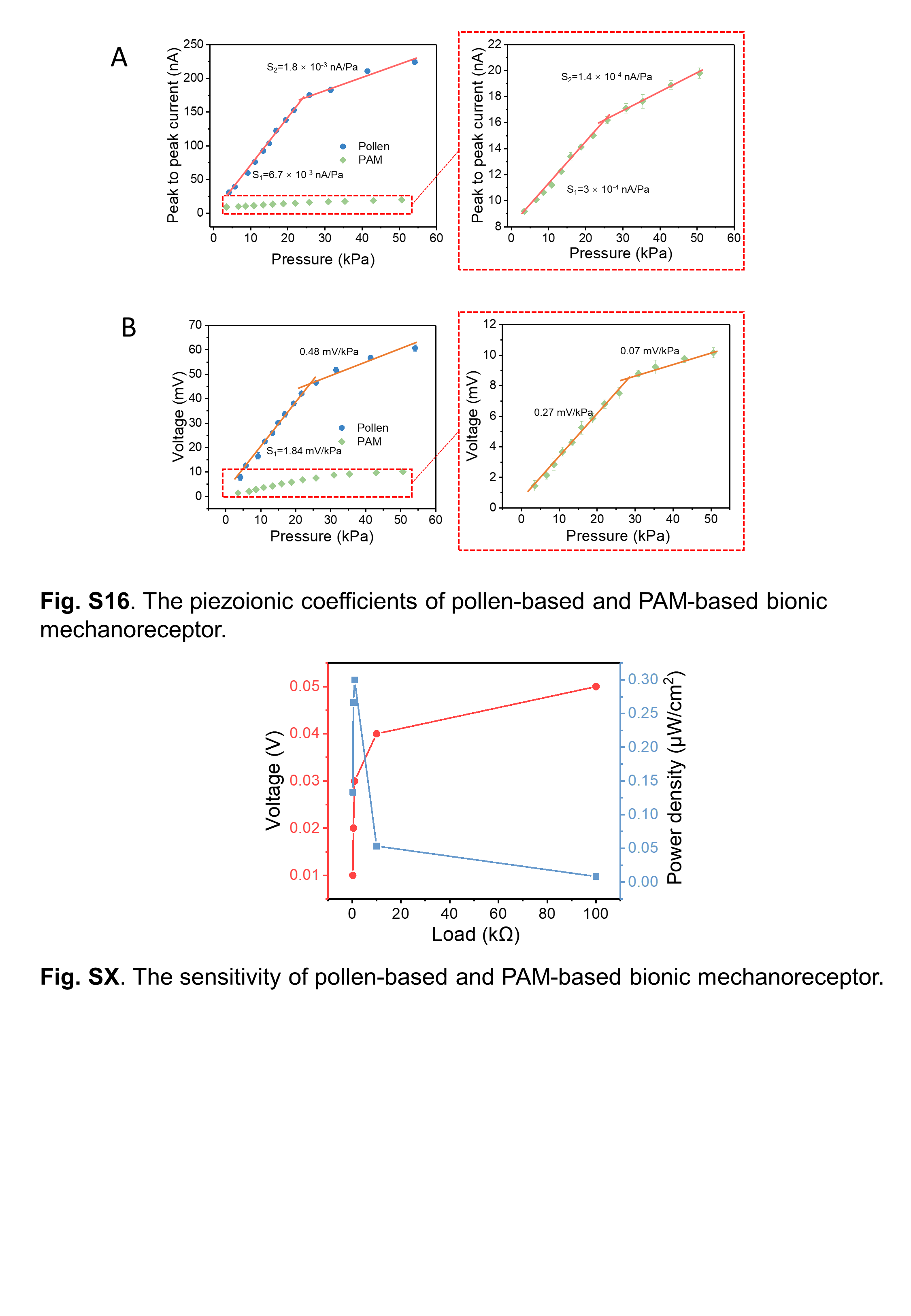


**Figure S16.**

The sensitivity of pollen-based and PAM-based bionic mechanoreceptor.


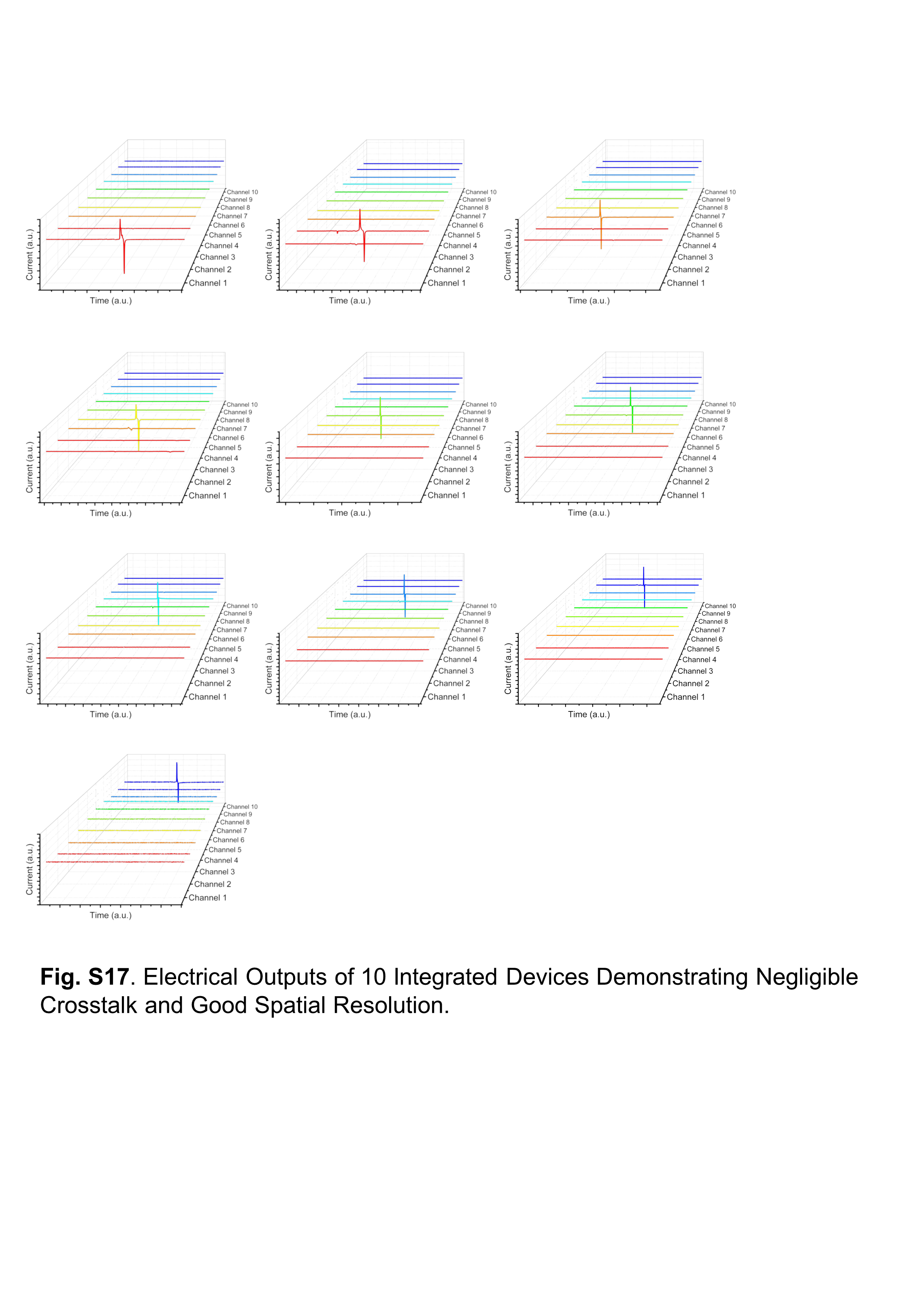


**Fig. S17**. Electrical Outputs of 10 Integrated Devices Demonstrating Negligible Crosstalk and Good Spatial Resolution.


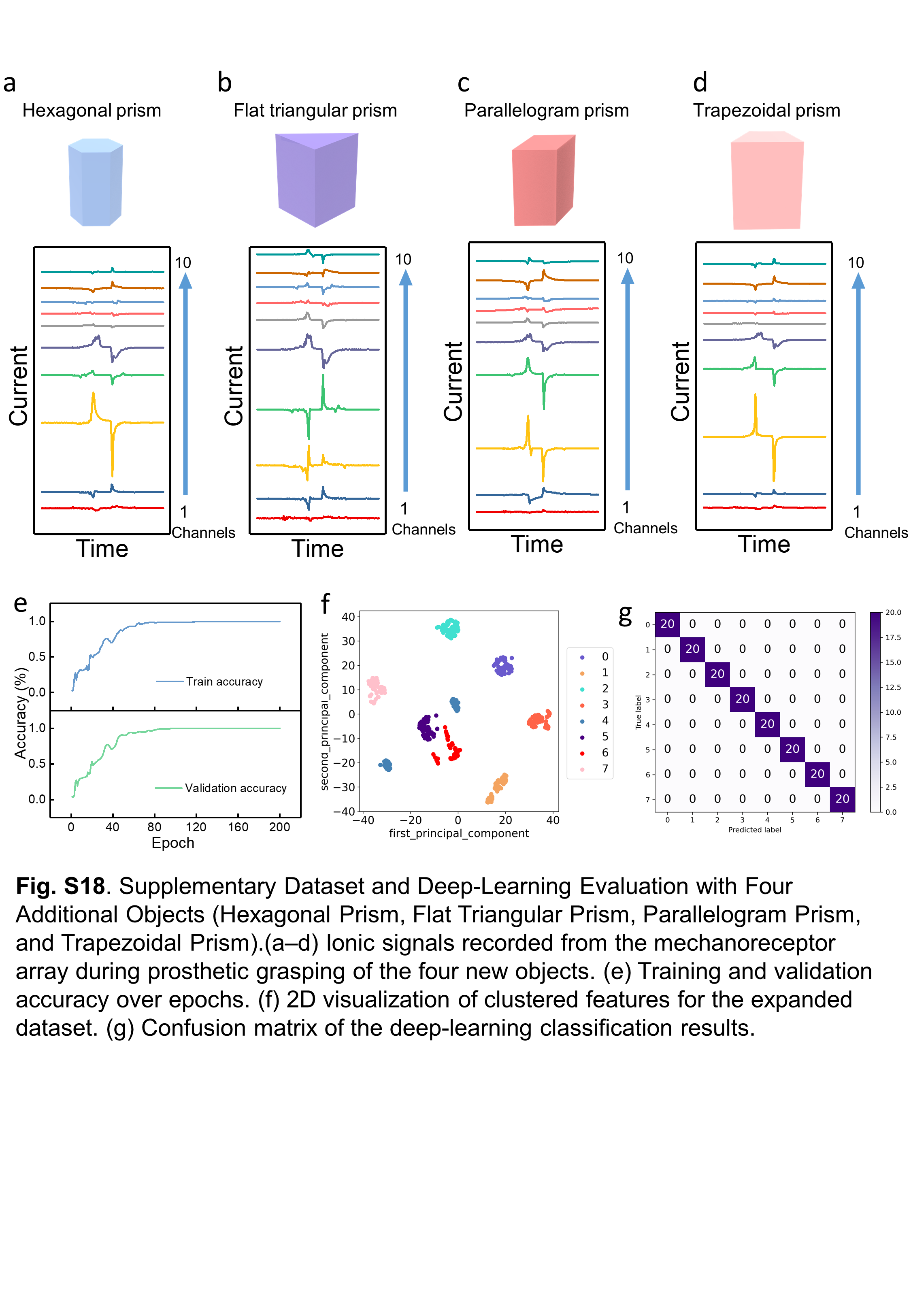


**Fig. S18**. Supplementary Dataset and Deep-Learning Evaluation with Four Additional Objects (Hexagonal Prism, Flat Triangular Prism, Parallelogram Prism, and Trapezoidal Prism).(a–d) Ionic signals recorded from the mechanoreceptor array during prosthetic grasping of the four new objects. (e) Training and validation accuracy over epochs. (f) 2D visualization of clustered features for the expanded dataset. (g) Confusion matrix of the deep-learning classification results.


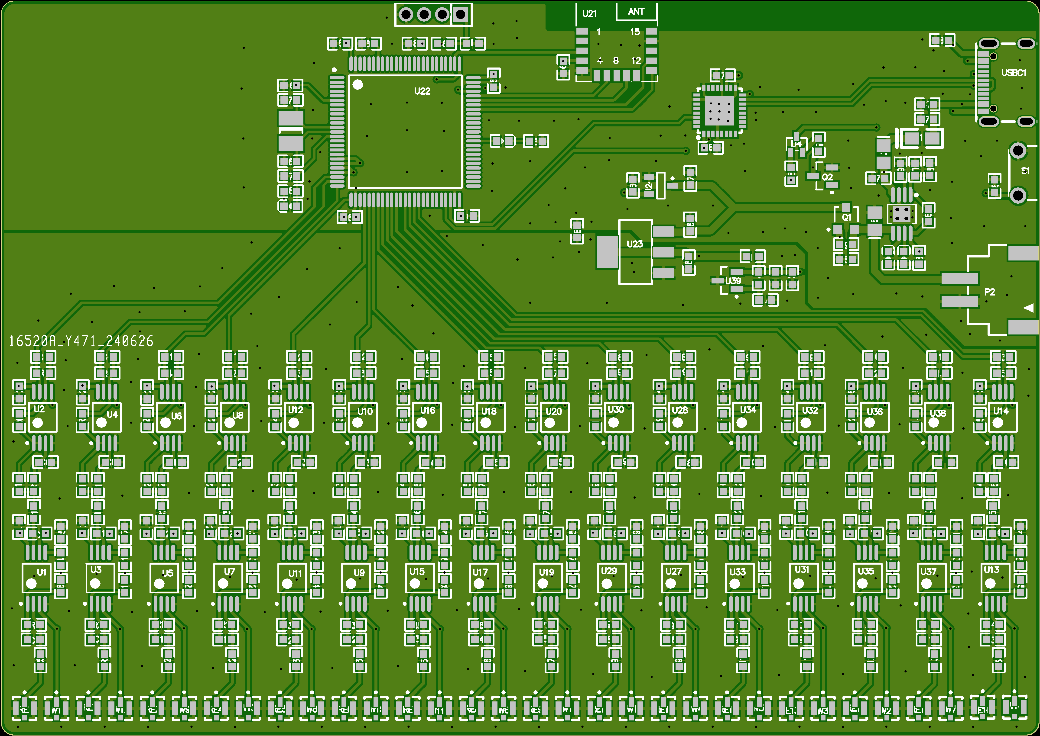


**Figure S19.**

The structure of PCB.


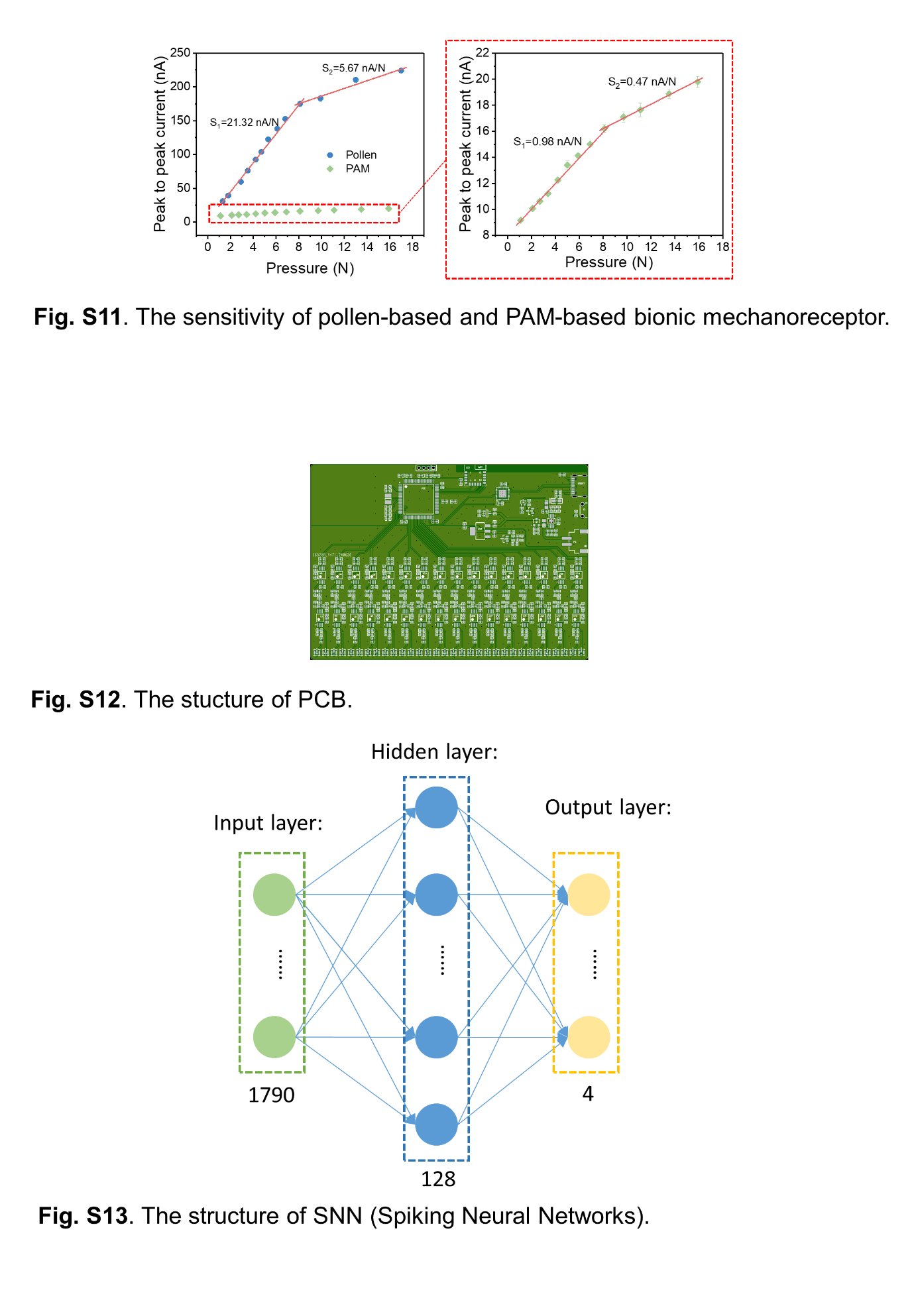


**Figure S20.**

The structure of SNN (Spiking Neural Networks).


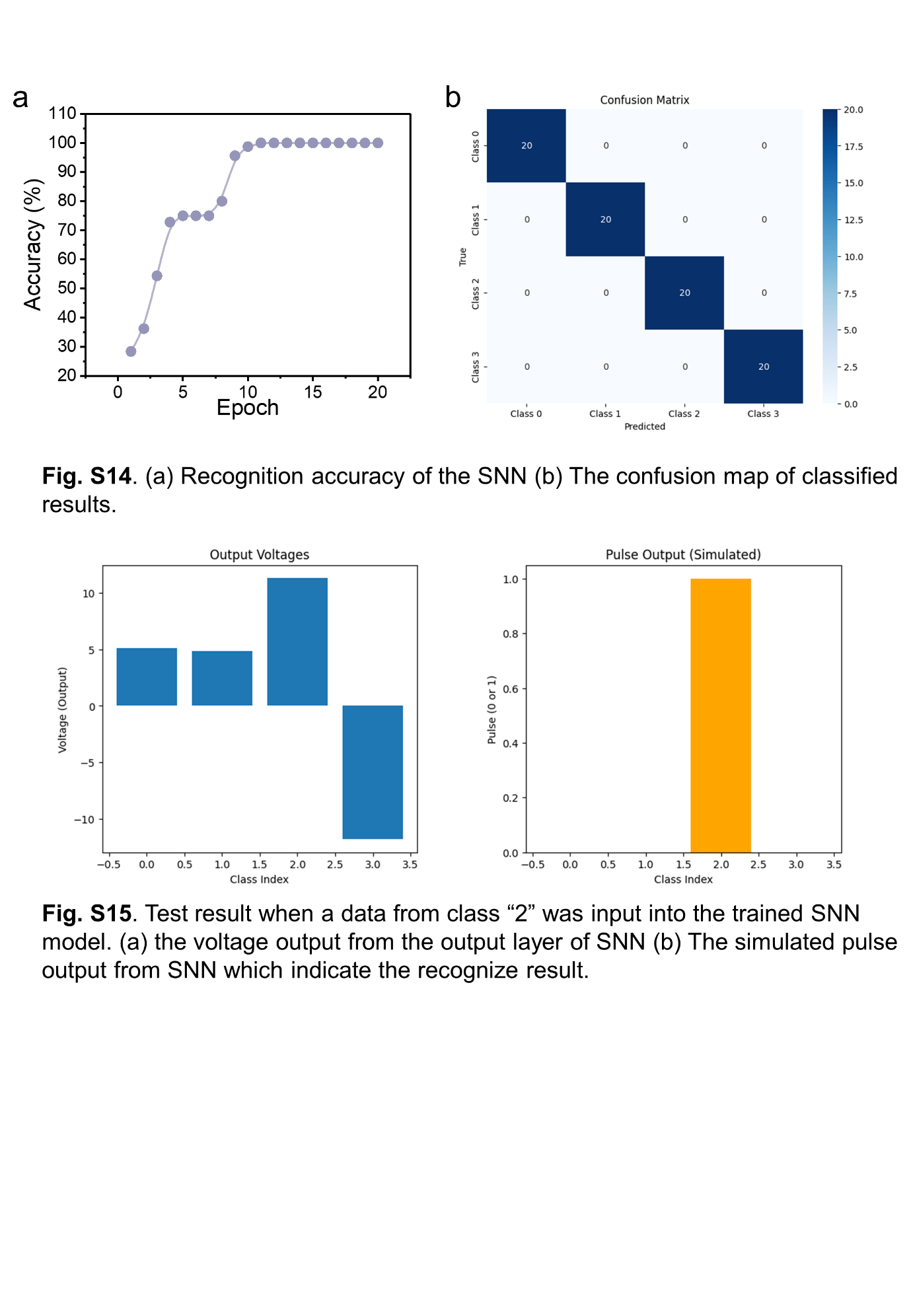


**Figure S21.**

**(a)** Recognition accuracy of the SNN; **(b)** The confusion map of classified results.


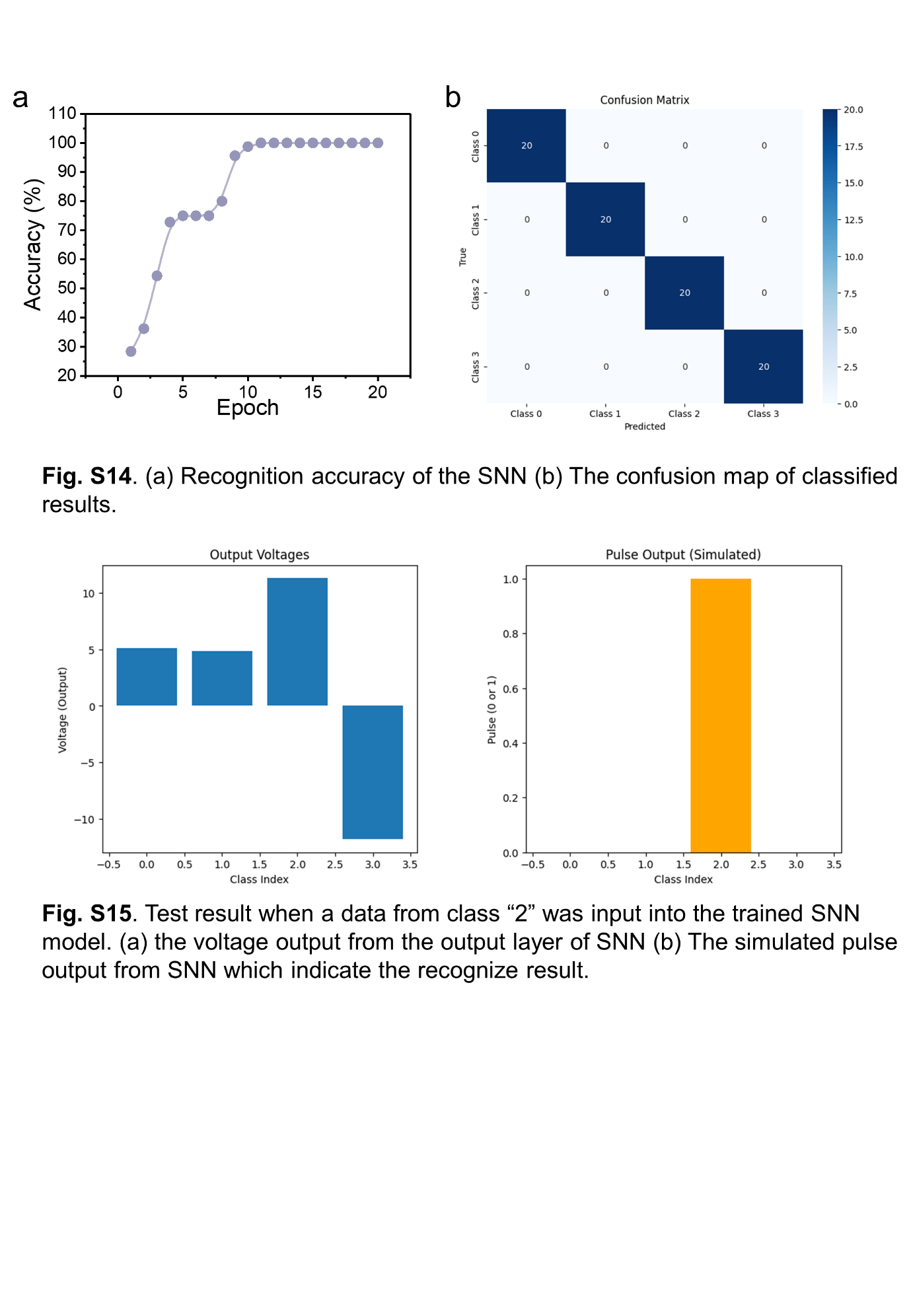


**Figure S22.**

Test result when a data from class “2” was input into the trained SNN model. **(a)** the voltage output from the output layer of SNN **(b)** The simulated pulse output from SNN which indicate the recognize result.


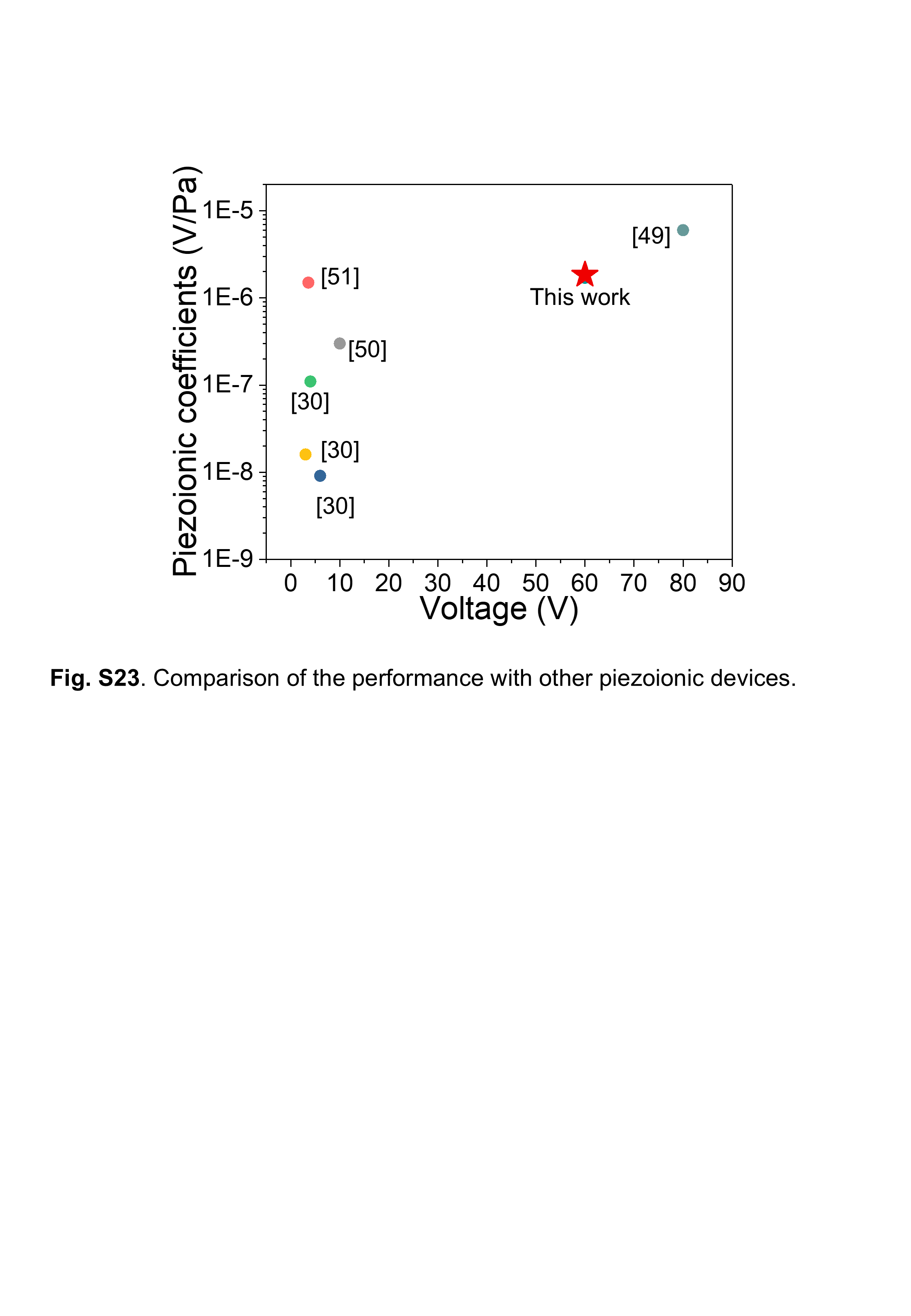


**Figure S23.**

Comparison of the performance with other piezoionic devices.

| Material | Voltage (mV) | Current (nA) | Piezoionic coefficients (V/Pa) | Ref |
| --- | --- | --- | --- | --- |
| PAAm/NaCl | 6 | 150 | 9.1×10^-9^ | [1] |
| PVDFHFP/LiTFSI | 4 | -- | 1.1×10^-7^ | [1] |
| P(AA-co-AAm) | 3 | -- | 1.6×10^-8^ | [1] |
| Conducting polymer | -- | -- | 5.0×10^-10^ | [2] |
| Ionomeric Polymer Metal Composites | -- | -- | 2.3×10^-10^ | [3] |
| SnSe Nanosheets-Double Network Hydrogel | 60 | 10^4^ | 1.7×10^-6^ | [4] |
| Ionic liquids and ionic plastic crystals | 80 | 80 | 6.0×10^−6^ | [5] |
| PVA/PEG/Gly/IL-TCPs | 10 | -- | 3×10^-7^ | [6] |
| Crown ether grafted PVA | 3.6 | -- | 1.5 ×10^−6^ | [7] |
| PAAM/NaCL | 10 | 12 | 2.7×10^−7^ | (This work) |
| Pollen modified PAAm | 60 | 150 | 1.84×10^−6^ | (This work) |

**Table S2.** **Comparison of the performance with other piezoionic devices.**

**Reference**

[1] Y. Dobashi, D. Yao, Y. Petel, T. N. Nguyen, M. S. Sarwar, Y. Thabet, et al., *Science* **2022**, *376*, 502.

[2] B. J. Akle, Virginia Polytechnic Institute and State University, **2005**.

[3] J. Torop, V. Palmre, M. Arulepp, T. Sugino, K. Asaka, A. Aabloo, *Carbon* **2011**, *49*, 3113.

[4] F. Li, X. Cai, G. Liu, H. Xu, W. Chen, *Advanced Functional Materials* **2023**, *33*, 2300701.

[5] W. Zhu, B. Wu, Z. Lei, P. Wu, *Adv. Mater.* **2024**, *36*, 2313127.

[6] S. Yuan, J. Bai, Y. Cao, S. Li, H. Zhu, T. Zhang, et al., *Advanced Functional Materials* **2025**, e15806.

[7] K. Yang, B. Li, Z. Ma, J. Xu, D. Wang, Z. Zeng, et al., *Angewandte Chemie International Edition* **2025**, *64*, e202415000.
